# Prognostic utility of pre‐biologic treatment correlates of childhood severe asthma exacerbation risk: Real world evidence

**DOI:** 10.1111/pai.70247

**Published:** 2025-12-03

**Authors:** Arthur H. Owora, Bowen Jiang, Yash Shah, Benjamin Gaston, Erick Forno

**Affiliations:** ^1^ Division of Pediatric Pulmonology, Allergy/Immunology and Sleep Medicine, Department of Pediatrics Indiana University School of Medicine Indianapolis Indiana USA; ^2^ Center for Biomedical Informatics Regenstrief Institute Indianapolis Indiana USA; ^3^ Translational Informatics, Biostatistics, Epidemiology, and Informatics (TIBE) Lab Indiana University School of Medicine Indianapolis Indiana USA

**Keywords:** biologics, childhood asthma, prognostic model, severe asthma exacerbations, treatment response

## Abstract

**Background:**

Biologics have been shown to substantially reduce the risk of severe asthma exacerbations (SAEs) among children with moderate or severe asthma; however, not all eligible patients who initiate biologic treatment experience a reduced risk of SAEs.

**Methods:**

Using a longitudinal study design, we analyzed data from 122 children with moderate/severe asthma treated with a biologic by pediatric subspecialists between December 2019 and December 2024. We used logistic and Cox proportional hazards models to identify and quantify the prognostic utility of clinical correlates of increased SAE risk among children with moderate or severe asthma prior to biologic treatment initiation.

**Results:**

Biologic agents (dupilumab, omalizumab, and mepolizumab) had differential effects on the risk of SAE depending on a child's sex (*p* = .024), age of treatment initiation (*p* = .010), lung function (% FEF_25‐75_ predicted: *p* = .034), and absolute neutrophils (*p* = .055). Dupilumab was associated with a higher risk of SAE among female patients. Omalizumab and mepolizumab were associated with a higher risk of SAEs among patients with elevated absolute neutrophils and younger age at treatment initiation. Consideration of these clinical parameters in a multivariable model improved the pre‐treatment prognostic accuracy of SAE risk by 23% compared to reliance on history of SAEs alone (0.86; 95% CI: 0.79, 0.89 vs. 0.63; 95% CI: 0.54, 0.72).

**Conclusion:**

Beyond biologic treatment eligibility, using routinely collected clinical parameters to stratify pre‐treatment SAE risk could improve prognostic accuracy and aid clinicians in tailoring therapy based on a patient's individual risk factors and likelihood of responding to a specific agent to maximize treatment effectiveness.

**Abbreviations**
AG: Andersen‐Gill
C‐ACT: childhood asthma control test
DCA: Decision curve analysis
ED: Emergency department
EHR: electronic health record
ICS: inhaled corticosteroids
ICS + LABA: inhaled corticosteroids in combination with long‐term acting beta‐agonist
IgE: serum immunoglobulin E
IUH: Indiana University Health
LASSO: least absolute shrinkage and selection operator
OCS: Oral corticosteroid
SABAs: short‐acting beta‐agonists
SAE: Severe Asthma Exacerbations
STROBE: Strengthening the Reporting of Observational Studies in Epidemiology
TRIPOD: Transparent Reporting of a Multivariable Prediction Model for Individual Prognosis or Diagnosis

**Key message**

Routinely collected clinical correlates of increased severe asthma exacerbation risk can improve pre‐biologic treatment prognosis accuracy to inform early targeted preventive interventions among at‐risk patients.

## 1 INTRODUCTION

For children with moderate‐to‐severe asthma characterized by Th2‐inflammatory markers, treatment with monoclonal antibodies (also known as “biologics”) has been shown to substantially reduce the risk of severe asthma exacerbations (SAEs)^1^, ^2^, ^3^, ^4^, ^5^, ^6^ defined as exacerbations that require hospitalization, emergency department (ED) admission, or systemic corticosteroids.^7^ However, not all eligible patients who initiate biologic treatment experience reduced risk of SAEs.^1^, ^2^, ^3^, ^4^, ^5^, ^6^ Moreover, eligibility defined by blood eosinophil counts, serum immunoglobulin E, or fractional exhaled nitric oxide (FeNO) does not necessarily predict treatment response. Despite the promise of biomarkers derived from metabolomics, proteomics, transcriptomics, and genomics, these approaches remain expensive, complex, unscalable, and therefore elusive in primary care settings.^8^, ^9^, ^10^ Among patients who initiate biologic treatment, the uncertainty regarding how and to what extent a patient's medical history may impact treatment prognosis stymies effective and proactive asthma disease management.

Although SAEs are often preceded by a period of poor asthma control,^11^ some research suggests that significant discrepancies may exist between asthma control and risk of SAE.^12^ Airway inflammation, a risk factor of SAEs, may persist in subjects with good clinical or physiological asthma control scores.^12^, ^13^ Therefore, clinicians need to be vigilant regarding the fluctuations in prognostic factors that can create discordance between risk of SAEs and asthma control when initiating new asthma treatments.^13^ After initiating a new treatment, there may be a window of opportunity during which additional timely preventive strategies that address underlying baseline SAE risk may help prevent future SAEs. A patient's electronic health records (EHR) data (e.g., previous diagnoses, medication history, laboratory results, asthma control, healthcare utilization) may offer an opportunity for assessing such underlying SAE risk to inform asthma management. Prognostic insights from such EHR data may enable clinicians to identify at‐risk individuals, enable earlier preventive interventions, and transition from reactive to proactive care. Although potentially useful, no studies to our knowledge have examined whether such routinely collected prognostic data can be leveraged to identify patients at increased risk of post‐biologic treatment SAEs before treatment initiation.

Therefore, the objective of our study is two‐fold: to identify and quantify the prognostic utility of routinely collected clinical correlates of increased SAE risk among children with moderate or severe asthma prior to biologic treatment initiation. We hypothesize that among children with moderate or severe asthma pre‐treatment correlates of SAE risk will have higher prognostic accuracy than current practice that relies on the history of SAEs alone for the prediction of post‐biologic treatment SAE risk.

## 2 METHODS

### 2.1 Study design and data sources

Study cohort was generated from the electronic health record (EHR) data warehouse at Indiana University Health (IUH) system affiliated institutions. IUH is a comprehensive healthcare system that provides primary and subspecialty care for over 1.2 million residents in Indiana. In addition, the Indiana Network for Patient Care (INPC) EHR databases which encompass over two‐thirds of the healthcare institutions in Indiana including IUH were used to obtain data on patient diagnoses, laboratory results, medication orders, and prescriptions from encounter‐specific records (inpatient, outpatient, and ED visits). Using the INPC databases minimized the likelihood of omitting asthma‐related encounters for the cohort that might have occurred outside the IUH system.

### 2.2 Study cohort

Our patient cohort included patients with asthma (ICD‐9/10‐CM 493.xx/J45.xx), aged 6–18 years, who were treated with an asthma biologic (omalizumab, mepolizumab, or dupilumab) by a pediatric pulmonologist or allergist in the Riley/IUH system between December 2019 and December 2024 (Figure S1). Biologics were prescribed, based on availability at the time treatment decisions were made, for children 6 years and older with severe, uncontrolled allergic asthma, specifically those who did not respond adequately to other treatments (e.g., inhaled corticosteroids [ICS] in combination with long‐term acting beta‐agonist [ICS + LABA]). For example, omalizumab was prescribed for children with elevated IgE, while mepolizumab and dupilumab were prescribed for children with severe eosinophilic asthma. To rule out patients who may have received previous asthma‐related care with biologics outside of IUH or the INPC, or who might have received biologics for other indications (e.g., atopic dermatitis), we excluded patients who had a biologic prescription before or on the same date as their first documented asthma diagnosis in our system, and those who started a biologic before age 6 years. EHR data was available for 2010–2024, so we extracted data for patients who had their first biologic prescriptions between 2012 and 2023, to allow at least 12 months of “post” biologic follow‐up.

### 2.3 Primary and secondary outcome

Our primary outcome was the occurrence of at least one acute severe asthma exacerbation (SAE), defined per ATS/ERS guidelines as an exacerbation that resulted in a hospitalization or ED visit during a 12‐month follow‐up period after biologic initiation.^7^ Oral corticosteroid (OCS) prescriptions not associated with a hospitalization or ER were excluded from the outcome definition to reduce the likelihood of outcome misclassification bias, since we could not determine from the EHR whether they were prescribed specifically for an acute asthma exacerbation. Our secondary outcomes included time to an incident and recurrent SAE events 12 months post biologic initiation.

### 2.4 Selected clinical parameters

Medication history included age at first exposure to any asthma‐related medication (ICSs, ICS + LABA, leukotriene modifiers, and short‐acting beta‐agonists [SABAs]) and all asthma‐related medications received 1 month prior to the index date.

Table S1 provides a summary of ICD9/ICD10 codes and key terms used to identify study covariates. These included age at first asthma diagnosis, sex, reported race, and history of parental asthma. Asthma‐related medication history included age at first prescription for ICS, ICS + LABA, or OCS. We calculated the burden of early‐childhood asthma risk factors using our validated passive digital marker (PDM) score^14^: As previously described, the PDM is constructed using EHR data at ≤3 years (a period of diagnostic uncertainty for asthma) that includes eczema, wheezing with or without a cold, pneumonia, bronchiolitis, and allergic sensitization and/or allergy reports.^14^ Lung function measures, body mass index (BMI), blood biomarkers (absolute neutrophil and eosinophil counts and %) and childhood asthma control test (C‐ACT) scores assessed and documented in EHR at least 60 days pre‐biologic initiation were considered for baseline covariate adjustment and variable selection in our analytic models.

### 2.5 Statistical analysis

#### 2.5.1 Correlates of SAE incidence and recurrence 12‐month post biologic initiation

We report descriptive statistics to summarize patient demographic and clinical characteristics at biologic initiation. We used Kaplan–Meier/cumulative incidence curves to characterize the time from a patient's first biologic medication to incident and recurrent SAE.

To identify risk correlates of SAE post‐biologic initiation, we examined the association between patient characteristics (demographic, allergy profile, pre‐biologic medication profile, and early childhood asthma burden [PDM]) and the SAEs as both a dichotomous and a possibly censored outcome (i.e., time from biologic initiation to SAE) using logistic and Cox proportional hazards regression models, respectively.

For our logistic models, the outcome variable was the occurrence of SAE within 12 months of biologic initiation (yes/no). Multivariable binomial logistic regression models were used to identify factors associated with increased odds of SAEs. For our Cox model, the outcome variable was time to a SAE event starting on the date of biologic initiation until a patient's first SAE, last 12‐month follow‐up date, or the end of study follow‐up (12/31/2024), whichever comes first. We also extended the Cox proportional hazards model (using a modified Andersen‐Gill [AG] recurrent event framework)^15^ to examine the relationships between covariates and the rate of recurrent SAE events (repeated SAE events within individuals) during the 12‐month follow‐up window after biologic treatment initiation. For both logistic and Cox models, we examined pairwise interaction terms to test whether the relationship between biologic agent and risk of SAE differed by patient characteristics. In the absence of modified effects (i.e., statistically significant interaction terms), multivariable models were used to determine to what extent patient characteristics were associated with increased (or earlier) odds of SAEs adjusted for potential confounders.

#### 2.5.2 Prognostic utility of SAE risk correlates after biologic treatment initiation

Prediction model selection and estimation: Prediction features/variables were selected based on statistical and clinical criteria. We used multivariable logistic regression with least absolute shrinkage and selection operator (LASSO) approach, which achieves selection of predictors (from a list of clinically relevant variables) by shrinking some coefficients to zero by setting a constraint on the sum of the absolute standardized coefficients.^16^ The optimal penalty under the LASSO approach was determined based on 5‐fold cross‐validation. The final LASSO model was evaluated by constructing a receiver‐operating characteristic (ROC) curve using a linear predictor. The linear predictor was calculated by summing up each LASSO penalized β‐coefficient multiplied by the corresponding variables' values. Youden's index was used to select a cutoff score that maximized sensitivity and specificity.

Internal validation: Model prognostic performance was internally validated using cross‐validation to assess the generalization of our prediction model's performance by repeatedly training and testing on different (5‐fold cross validation) data partitions. Bootstrap resampling (1000 resamples) was used to examine sampling variability and assess our prediction model's optimism (including bias‐corrected estimates) for each performance metric (e.g., sensitivity and specificity).^17^

Clinical utility: Decision curve analysis (DCA)^18^ was used to evaluate the potential benefits and harms of using our prognostic model across a range of threshold probabilities, which represent a clinician's willingness to act on a predicted risk. Five‐fold cross‐validation was used to assess the robustness of our prognostic model performance. By plotting net benefit against predicted risk thresholds, DCA was used to determine if our prognostic model improves clinical decision‐making compared to standard approaches like “treat all” or “treat none”. Improvements in prognostic accuracy (vs history of SAE – a proxy for current practice) were summarized using a continuous net reclassification improvement index, and net benefit.^19^ Kaplan–Meier analysis was used to quantify the impact of risk classification using our prognostic model for earlier detection of patients at risk of SAE before biologic treatment initiation.

Sensitivity analyses: Our final prediction model performance was examined in subgroups defined by history of SAE, OCS use, and biologic agent to assess the robustness of our model predictions under different case mix scenarios.

Post hoc power estimation: Table S2 summarizes the post‐hoc sample size/power considerations for the prognostic model (Cox proportional hazards model and logistic model).^20^ Briefly, if we assume a constant event rate of 0.15 and an average follow‐up time of 1.0 years, with a Cox proportional hazards model containing a three‐level categorical variable (3 biologic agents) and an expected R‐squared value of .15, the required sample size would be *N* = 110 patients, with an event per predictor (EPP) of 8.66. For a logistic model (with similar considerations and an expected C‐statistic of 0.8), a sample size of 110 was adequate to minimize overfitting and for the absolute difference between the apparent and adjusted R‐squared, but potentially underpowered to yield precise estimates of the average outcome risk.

Two‐tailed *p*‐values <.05 were considered statistically significant. All data analyses were conducted using R version 4.4.3. We followed the Strengthening the Reporting of Observational Studies in Epidemiology (STROBE) reporting guidelines for cohort studies and the Transparent Reporting of a Multivariable Prediction Model for Individual Prognosis or Diagnosis^21^ (TRIPOD) guidelines. The Indiana University institutional review board approved all study procedures with a waiver of informed consent for the use of retrospective data (IRB#15873).

## 3 RESULTS

### 3.1 Participant Characteristics

Table 1 summarizes the demographic and clinical characteristics of the study cohort (*n* = 122): briefly, 50.8% were female, 60.7% were Black, and 4.1% were Hispanic/Latino. Cohort patients had an average age of 3.1 years (SD = 3.6 years) at their incident asthma diagnosis and 10.5 years (SD = 3.7) at biologic initiation. Treatment history, lung function measures, BMI, blood biomarkers (absolute neutrophil and eosinophil counts and %) and asthma control test (ACT) scores did not differ by biologic type (Table S3). Dupilumab was the most frequently prescribed biologic (63.1%) followed by omalizumab (27.0%) and mepolizumab (9.8%). Approximately 54% of patients started biologics aged 6–11, with the remaining 46% starting after age 12.

**TABLE 1:** 12‐month cumulative incidence and recurrence of severe asthma exacerbations (SAEs) post‐biologic initiation.

Patient characteristics	Overall	SAE incidence (≥1)	*p* Value^b^	SAE recurrence (≥2)	*p* Value^b^
Overall	**122**	19 (15.6%)		14 (11.5%)	
Biologic agent					
Dupilumab	77 (63.1%)	4 (5.2%)	**<.001**	4 (5.2%)	.**008**
Omalizumab	33 (27.0%)	10 (30.3%)		6 (18.2%)	
Mepolizumab	12 (9.8%)	5 (41.7%)		4 (33.3%)	
Sex					
Female	62 (50.8%)	11 (17.7%)	.502	9 (14.5%)	.284
Male	60 (49.2%)	8 (13.3%)		5 (8.3%)	
Race					
White	43 (35.2%)	4 (9.3%)	.239	4 (9.3%)	.768
Black/African American	74 (60.7%)	15 (20.3%)		10 (13.5%)	
Others^a^	5 (4.1%)	0 (0.0%)		0 (0.0%)	
Ethnicity					
Non‐Hispanic/Latino	117 (95.9%)	17 (14.5%)	.172	12 (10.3%)	.100
Hispanic/Latino	5 (4.1%)	2 (40.0%)		2 (40.0%)	
PDM risk					
Low	34 (27.9%)	3 (8.8%)	.442	2 (5.9%)	.511
Moderate	33 (27.0%)	6 (18.2%)		4 (12.1%)	
High	55 (45.1%)	10 (18.2%)		8 (14.5%)	
Asthma diagnosis					
Mean Age (SD) in years	3.1 (3.7)	1.7 (3.1)	.**006**	**1 (2)**	.**003**
Biologic initiation					
Mean Age (SD) in years	11.1 (3.3)	10.6 (3.3)	.419	11 (3)	.397
Biomarkers					
Mean (SD) Eosinophil (%)	6.1 (3.9)	5.9 (4.5)	.711	7 (5)	.596
Mean (SD) Neutrophil (%)	55 (13)	60 (15)	.133	56 (11)	.431
History (1‐year pre‐biologic initiation)					
ICS + LABA^C^					
No	14 (11.5%)	1 (7.1%)	.694	0 (0.0%)	.366
Yes	108 (88.5%)	18 (16.7%)		14 (13.0%)	
SAE					
No	67 (54.9%)	5 (7.5%)	.**006**	3 (4.5%)	.**007**
Yes	55 (45.1%)	14 (25.5%)		11 (20.0%)	

*Note:* Bold values represent statistically significant differences (*p* < 0.05).

^a^ Others in Race: Asian 1 (0.8%); not reported 4 (3.3%).

^b^ *p* values are based on covariate comparison between patients with vs. without incident SAEs.

^c^ ICS + LABA: budesonide‐formoterol 28 (23.0%); fluticasone‐salmeterol/vilanterol 69 (56.6%); mometasone‐formoterol 20 (16.4%).

### 3.2 Cumulative incidence and recurrence of SAE Post‐biologic initiation (12‐month follow‐up)

**FIGURE 1:**
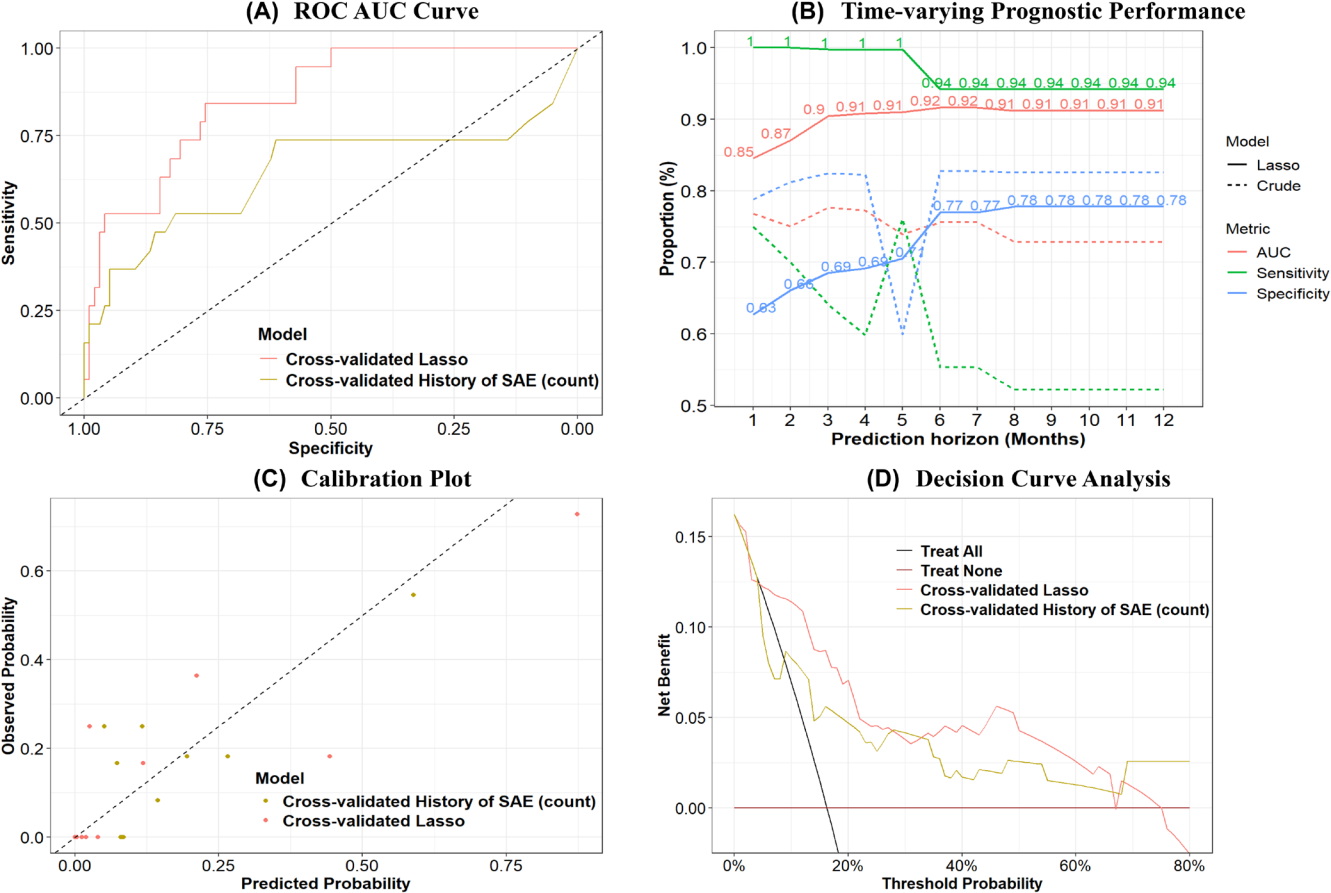
Cumulative Incidence and Recurrence of SAEs 12‐months post‐biologic treatment initiation. Overall Cumulative Incidence and recurrence of SAEs (A); biologic‐specific cumulative Incidence and recurrence of SAEs (B).

Overall, 19 (15.6%) children had at least one SAE within 12‐months of biologic treatment initiation (Table 1 and Figure S2). Among the 19 patients who experienced an incident SAE during follow‐up (12‐months post biologic initiation), 84% had a recurrent event within 12‐months (Figure 1A). A shorter time to SAE was observed for patients taking omalizumab and mepolizumab than dupilumab (Figure 1B, Gray's test: *p* < .01). Conversely, time to a recurrent SAE event was marginally shorter for dupilumab than omalizumab or mepolizumab (Figure 1B, Gray's test: *p* = .09).

**FIGURE 2:**
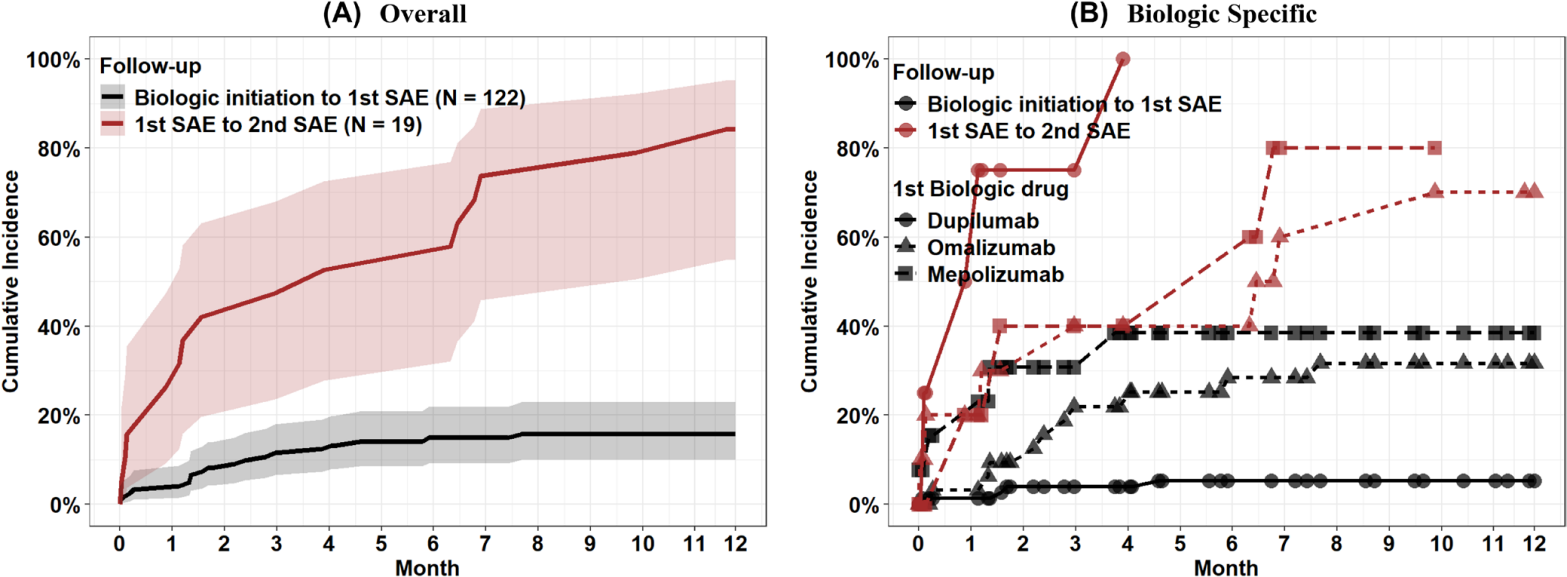
Correlates of childhood Severe Asthma Exacerbations (SAEs) risk differ by biologic agent. Logistic model results of effect modification by biologic type including 95% confidence interval bands representing the upper and lower bounds (A); continuously and discretely colored survival area plots displaying different estimates of the FEF_25–75_ predicted (%) values on time to SAE summarized as a survival probability (B).

Multivariable regression models were used to identify patient characteristics associated with higher (binomial model: Table 2 and Table S4) and earlier (Cox model: Table 2 and Table S5) odds of SAEs 12‐months after biologic initiation. To test the hypothesis that biologic agent modified the SAE risk corelates, we examined the significance of pairwise interaction terms involving biologic agent and patient characteristics. Biologic agent modified the effects of sex (*p* = .024), age of biologic treatment initiation (*p* = .010), lung function (% FEF_25‐75_ predicted: *p* = .034), and absolute neutrophils (*p* = .055) on risk of SAE (Figure 2). Dupilumab was associated with higher risk of SAE among female patients. Omalizumab and mepolizumab were associated with higher risk of SAEs among patients with elevated absolute neutrophils and younger age at treatment initiation. However, because the absolute risk (probability) differences had overlapping 95% confidence intervals by biologic agent (Figure 2), we also report the main/simple effects.

**TABLE 2:** Demographic and Clinical Correlates of Severe Asthma Exacerbations (SAEs) Post‐Biologic Initiation Summarized by Odds Ratios and Hazard Ratios (including 95% CIs).

Patient characteristics	Odds ratio [OR] (95% CI)		Hazard ratio [HR] (95% CI)	
Overall	Crude OR	Adjusted OR^a^	Crude HR	Adjusted HR^a^
Biologic agent				
Dupilumab	Reference	Reference	Reference	Reference
Omalizumab	**8.33 (2.51, 33.0)**	**6.70 (1.71, 30.9)**	**6.59 (2.07, 21.0)**	**6.49 (1.79, 23.5)**
Mepolizumab	**12.5 (2.74, 62.2)**	**8.89 (1.48, 55.1)**	**10.4 (2.78, 38.6)**	**7.47 (1.52, 36.6)**
Sex				
Female	Reference	Reference	Reference	Reference
Male	0.70 (0.25, 1.87)	0.57 (0.20, 1.56)	0.70 (0.28, 1.74)	0.60 (0.24, 1.49)
Race				
White	—	—	—	—
Black/African American	2.48 (0.83, 9.19)	2.10 (0.66, 8.15)	2.23 (0.74, 6.73)	1.86 (0.59, 5.92)
Others^a^	—	—	—	—
Ethnicity				
Non‐Hispanic/Latino	Reference	Reference	Reference	Reference
Hispanic/Latino	3.73 (0.47, 24.1)	3.35 (0.40, 23.0)	3.27 (0.75, 14.2)	3.24 (0.72, 14.6)
PDM risk				
Low	Reference	Reference	Reference	Reference
Moderate	2.23 (0.53, 11.4)	0.28 (0.02, 4.49)	2.12 (0.53, 8.49)	0.39 (0.03, 4.67)
High	2.25 (0.62, 10.7)	0.09 (0.00, 6.06)	2.14 (0.59, 7.77)	0.17 (0.00, 7.03)
Asthma diagnosis				
Mean Age (SD) in years	**0.83 (0.64, 0.99)**	0.83 (0.59, 1.07)	0.84 (0.68, 1.03)	0.83 (0.63, 1.11)
Pre‐school age: <4 years	**4.32 (1.14, 28.2)**	3.90 (0.27, 83.1)	3.81 (0.88, 16.5)	**17.9 (1.17, 272)**
Biologic initiation				
Mean Age (SD) in years	0.94 (0.81, 1.10)	0.99 (0.83, 1.16)	0.95 (0.82, 1.09)	0.98 (0.84, 1.15)
Biomarkers				
Mean (SD) Eosinophil (%)	0.99 (0.87, 1.12)	0.99 (0.86, 1.11)	0.99 (0.88, 1.11)	0.99 (0.88, 1.11)
Mean (SD) Neutrophil (%)	1.03 (0.99, 1.07)	1.02 (0.99, 1.06)	1.69 (0.47, 6.03)	1.02 (0.99, 1.05)
Absolute Neutrophils (K/μL)	**1.24 (1.02, 1.51)**	**1.23 (1.01, 1.51)**	**1.19 (1.02, 1.39)**	**1.19 (1.02, 1.39)**
History (1‐year pre‐biologic initiation)				
ICS + LABA				
No	Reference	Reference	Reference	Reference
Yes	2.51 (0.45, 47.2)	1.91 (0.30, 37.3)	2.45 (0.33, 18.3)	1.88 (0.24, 14.7)
SAE				
No	Reference	Reference	Reference	Reference
Yes	**4.24 (1.49, 14.0)**	**3.49 (1.17, 12.0)**	**3.73 (1.34, 10.4)**	**3.15 (1.09, 9.06)**

*Note:* Bold values represent statistically significant differences (*p*<0.05).

^a^ Models adjusted for PDM score, first biologic type, number of SAEs in the year prior to first biologic exposure, and baseline predicted FEV_1_/FVC (%), age at first asthma diagnosis, and age at biologic initiation as needed.

Compared to dupilumab, omalizumab, and mepolizumab were associated with higher odds of SAEs (Table 2). AG models showed the risk of SAE recurrence was higher among patients treated with omalizumab (Hazard ratio: 3.59; 95% CI:1.78, 7.25) and mepolizumab (Hazard ratio: 7.28; 95% CI: 3.50, 15.15) than dupilumab even after adjusting for differences in SAE history, age of asthma diagnosis, and early childhood risk factors (PDM).

Children with elevated baseline absolute neutrophils, a pre‐school asthma diagnosis, and a history of SAE(s) had higher and earlier odds of SAEs (Table 2). Treatment history (ICS + LABA), BMI, asthma control scores, and absolute (or %) eosinophils at the time of biologic initiation were not associated with increased (Table S3) or earlier (Table S5) odds of SAEs.

### 3.3 Prognostic utility of pre‐biologic clinical/medication history for treatment prognosis

**FIGURE 3:**
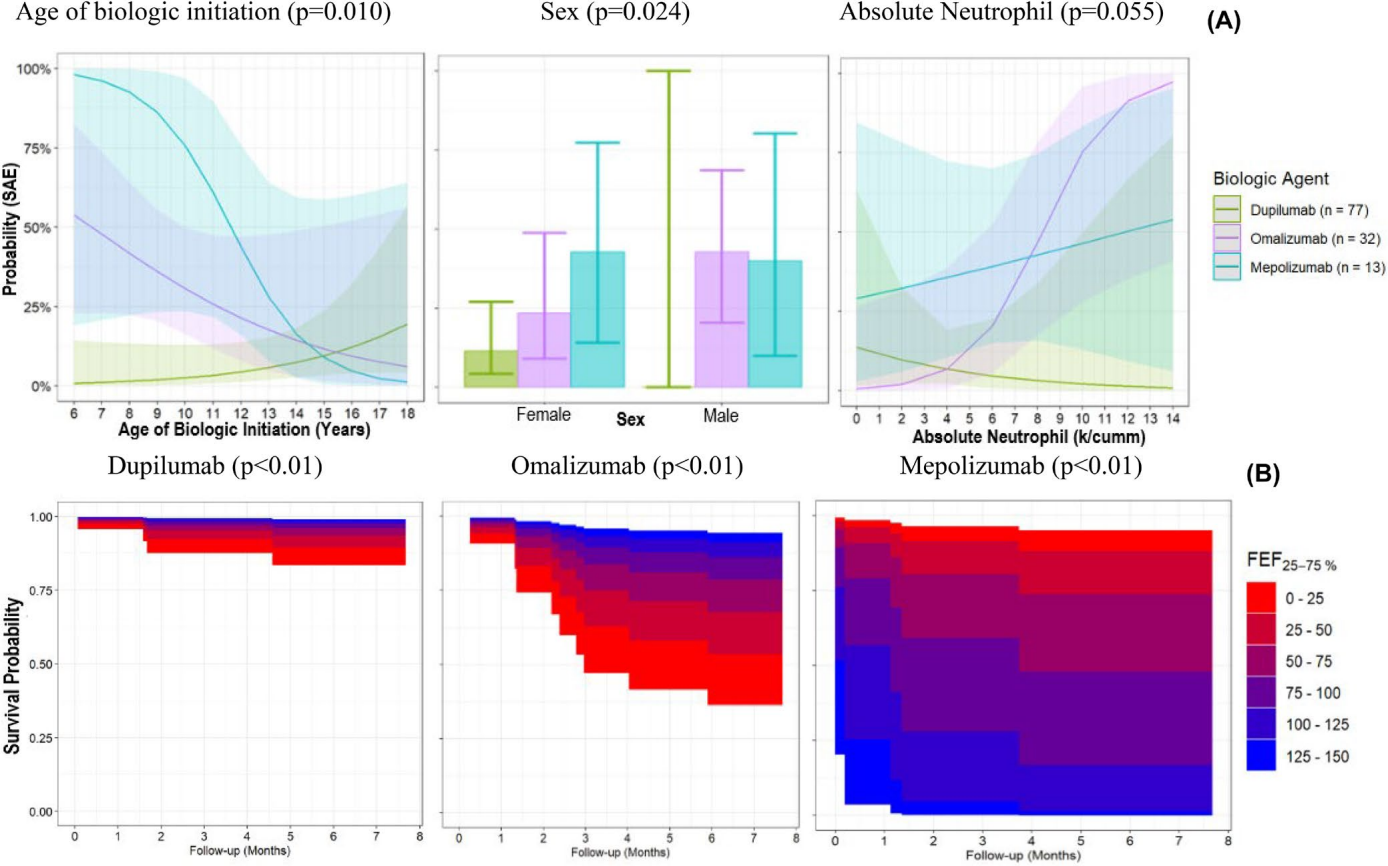
Prognostic performance of SAE prediction models (corrected for optimism) post‐biologic initiation. Comparison of the AUCs between the LASSO and crude Model (A); AUC comparisons between LASSO and crude model across different prediction horizons (B); comparison of Calibration plots between LASSO and crude model (C); net‐benefit comparisons across different risk threshold between the LASSO and crude model (D).

Compared to a crude model based on prior SAEs (a proxy for current practice), our LASSO‐derived multivariable model including the ethnicity, pre‐school age asthma, type of biologic, lung function, and history of SAEs had higher discrimination (Logistic model: Figure 3A; Cox model: Figure 3B), calibration (Figure 3C), and net benefit (Figure 3D) for the prediction of SAEs 12 months post biologic initiation (Table 3; Table S5). The LASSO model had an EPP of 2.7 while the crude model had an EPP of 19. There was a strong correlation (Figure S3) between selected LASSO predictors and risk factors associated with increased risk of post‐biologic treatment SAEs. The LASSO model had a higher prognostic accuracy (0.86; 95% CI: 0.79, 0.89) than the history of SAEs alone (0.63; 95% CI: 0.54, 0.72) for early detection of patients at risk of SAEs. Decision curve analysis showed that the prediction model was clinically useful for thresholds over 6% adjusted for model optimism (cross‐validated model: Figure 3D). The LASSO model (including reduced LASSO model [EPP of 3.8]) had a higher net benefit than the crude model above a threshold probability of 15%–60% with a net reclassification index (continuous: 1.19; 95% CI: 0.82, 1.56; categorical: 0.50; 95% CI: 0.20, 0.80) that demonstrated improved discriminative performance. The prognostic accuracy of different predictor configurations of the multivariable model is provided in Figure S4. We constructed a nomogram (Figure S5 – Logistic model) to provide a visual representation of the final LASSO model that includes the relative importance (by the number of points attributed) of each predictor to aid clinicians in conducting individualized risk assessments.

**TABLE 3:** Prognostic performance of the LASSO vs. crude (i.e., history of SAEs) Logistic models.

Model	Accuracy	Sensitivity	Specificity	PPV	NPV	AUC	Cut‐off probability
Observed (unadjusted for model optimism)							
LASSO	0.86	0.89	0.86	0.55	0.98	0.93	.20
LASSO+	0.85	0.89	0.84	0.53	0.98	0.93	.20
LASSO−	0.83	0.79	0.84	0.48	0.95	0.84	.18
SAE History (Count)	0.77	0.53	0.82	0.36	0.90	0.73	.19
SAE History (Yes/No)	0.63	0.74	0.60	0.26	0.92	0.67	.17
Cross‐validated (Adjusted for model optimism)							
LASSO	0.77	0.84	0.76	0.40	0.96	0.86	.11
LASSO−	0.79	0.68	0.82	0.42	0.93	0.77	.17
SAE History (Count)	0.63	0.74	0.61	0.27	0.92	0.65	.12
SAE History (Yes/No)	0.62	0.74	0.60	0.26	0.92	0.54	.16

*Note*: Sensitivity: probability that a predicted result will be positive when the disease is present (true‐positive rate). Specificity: probability that a predicted result will be negative when the disease is not present (true‐negative rate). Positive predictive value (PPV): probability that the disease is present when the predicted result is positive. Negative predictive value (NPV): probability that the disease is not present when the predicted result is negative. Accuracy: overall probability that a patient is correctly classified = Sensitivity × Prevalence + Specificity × (1 − Prevalence). LASSO Model: log(*p*/(1‐*p*)) = 8.66 + 2.95Hispanic + 3.42Preschool‐asthma + 2.61Omalizumab + 2.75Mepolizumab‐4.32OCS‐history + 0.58SAE‐history‐0.13FEV_1_ predicted (%). LASSO+ Model: log(*p*/(1‐*p*)) =8.83 + 2.95Hispanic + 3.44Preschool‐asthma+2.62Omalizumab + 2.77Mepolizumab‐4.32OCS‐history+0.58SAE‐history‐0.14FEV_1_predicted (%)‐0.01Absoulute neutrophil. LASSO‐ Model: log(*p*/(1‐*p*)) =8.06 + 0.0006PDM + 1.90Omalizumab + 2.18Mepolizumab + 0.63SAE history‐0.13FEV_1_ predicted (%). Crude Model: SAE History (yes/no): log(*p*/(1‐*p*)) = −2.47 + 1.44SAE history. Crude Model: SAE History (count): log (*p*/(1‐*p*)) = −2.50 + 0.69SAE history.

Abbreviation: AUC, area under the curve.

Internal validation of the LASSO model with 5‐fold cross‐validation (Figure S4) and bootstrapping (Figure S6) showed modest generalizability, stability, and optimism of model performance, respectively. For example, sensitivity had an estimated bias of 0.04 with a bias‐corrected value of 0.85 while specificity had an estimated bias of <0.01 with a bias‐corrected value of 0.86. Risk score stratified analyses showed that the odds of SAEs occurred earlier among children classified in the high (vs. low) risk strata by our prognostic model (Figure S4); higher hazards were observed among patients classified as high risk compared to the use of the history of SAEs (Figure S2).

Subgroup analyses (Table S6) defined by history of SAE showed stable accuracy (AUC: 0.89) despite a reduction in study sample size (and statistical power). Biologic agent specific prognostic accuracy was more variable (AUC: dupilumab – 0.66, omalizumab – 0.75, and Mepolizumab – 0.77) reflecting potential differences in case mix. However, overall model prognostic accuracy was relatively unchanged (AUC: 0.87) among patients who continued biologic treatment into the 2nd year of follow‐up (i.e., had at least one completed biologic prescription in the 2nd year of follow‐up).

## 4 DISCUSSION

Our findings show that biologic agents may be differentially associated with pre‐treatment correlates (age, sex, lung function, and absolute neutrophils) of SAE risk after treatment initiation. Among treatment‐eligible patients, the consideration of pre‐treatment correlates of SAE risk in a multivariable prognostic model is associated with higher prognostic accuracy and clinical utility for early identification of children at risk of post‐treatment SAEs than the current practice of considering the history of SAEs alone. In addition to a selected biologic agent (a surrogate marker of the suspected etiologic pathway), the consistency of pre‐school age, lung function, neutrophils, and history of SAEs, as inter‐related SAE risk (i.e., increased probability of SAE) and prognostic (i.e., predicting future SAE incidents) factors underscore their etiologic and prognostic relevance for clinical decision‐making.

Compared to dupilumab, the higher odds of SAEs among patients treated with omalizumab and mepolizumab underscore the treatment challenge associated with biologic agents that target a limited spectrum of asthma disease pathways.^22^ The higher risk of SAEs among younger patients with elevated IgE (targeted by omalizumab), eosinophils (targeted by mepolizumab), and relatively higher neutrophils (compared to patients treated with dupilumab) is consistent with previous studies that suggest biologics that target multiple pathways (dupilumab or tezepelumab) might be a better option for patients with unclear etiology or endotypes.^23^ Notably, treating patients with non‐eosinophilic inflammatory pathways is clinically challenging, though ongoing research into new therapies like Bcl‐2 inhibitors and agents targeting neutrophil chemoattractants shows some promise but RCTs in pediatric populations with asthma are needed to confirm their efficacy.^24^

During early childhood, asthma‐related morbidity may cause dysfunctional airways remodeling (linked to poor lung function) that may be associated with a higher risk of SAE even after the initiation of disease‐modifying treatment (e.g., biologics).^25^ Therefore, the pre‐school years are a critical time for intervention (e.g., treatment with ICS) to reduce the risk of such asthma‐related morbidity. Evidently, some degree of pre‐school age asthma misdiagnosis may occur,^26^ but this does not outweigh the costs associated with a delayed or undertreated asthma disease.^27^ Among children with persistent asthma symptoms at school age (6+ years), an earlier age of biologic (dupilumab) treatment initiation could be more effective in modifying the natural course of disease towards a reduced risk of SAEs.

Our results are consistent with previous studies^28^, ^29^, ^30^ that show a history of SAEs is a strong predictor of post‐treatment SAE risk in children regardless of biologic agent, age, asthma control, and medication history. Moreover, the persistently heightened risk of recurrent SAEs even after biologic treatment initiation underscores the need for earlier consideration of additional SAE preventive strategies among at‐risk patients especially those with a pre‐school age asthma diagnosis. Such considerations may need to be made early, even while the patient is being followed on biologics for 4–6 months before alternative biologic agents are considered.^31^

Our finding of a differential biologic response by sex is consistent with a recent study that observed innate differences in immune cells along the airways between male and female patients with severe asthma that could impact biologic treatment response.^32^ However, absent a randomized controlled study design, the potential impact of unmeasured confounding on observed results cannot be ruled out. The higher risk of SAE among female (vs male) patients treated with dupilumab needs validation in future larger (higher‐powered) randomized controlled trial studies especially among children who meet criteria for multiple biologics.

This study has some limitations. While our study was relatively large for a pediatric cohort on biologics, our analysis was likely somewhat underpowered to develop a multivariable prognostic model with more than two parameters and detect independent effects of biologic treatment on SAE risk. Therefore, our prognostic insights and measures of association should be interpreted cautiously and warrant replication in future studies. However, the consistency of our prognostic model results with different configurations of the highly correlated predictors even when the set of included predictors was varied across bootstrap samples suggests robust stability of individual‐level predictions. The effect of unmeasured confounders after treatment initiation (e.g., changes in biologic treatment, medication adherence or environmental risk factors) on the risk of SAEs cannot be excluded. Biologic use may be overestimated because medication orders do not indicate that the medication was taken as prescribed. Miscoding or missing data in emergency department or hospitalization EHR data systems may occur. However, these coding or omission errors would be consistent across comparison groups and therefore should not produce substantial non‐differential misclassification bias (i.e., bias towards the null). Given our relatively small sample size, we had low statistical power to detect biologic agent‐specific risk factors of SAE risk. The selection of Hispanic/Latino ethnicity as a prognostic (but not risk) factor in our models warrants cautious inference because it may reflect the impact of socio‐economic disadvantage in the absence of a direct evaluation of socio‐economic variables. Despite the small sample size, our internal validation based on bootstrapping (1000 resamples) and 5‐fold cross‐validation shows promising results for prognostic model stability and generalizability; however, external validation is still warranted.

In summary, the use of routinely collected clinical parameters could improve the accuracy of pre‐biologic treatment prognosis and aid clinicians in tailoring therapy based on a patient's individual risk factors and likelihood of responding to a specific agent to maximize treatment effectiveness. Furthermore, our findings emphasize the need for earlier preventive intervention among patients at risk of incident SAE post‐biologic treatment initiation to attenuate the risk of SAE recurrence. However, external validation and further investigation are needed to assess the efficacy of our prognostic model to inform care management that results in SAE risk reduction.

## AUTHOR CONTRIBUTIONS

**Arthur H. Owora:** Conceptualization; writing – original draft; methodology; funding acquisition; writing – review and editing. **Bowen Jiang:** Formal analysis; writing – review and editing. **Yash Shah:** Formal analysis; writing – review and editing. **Benjamin Gaston:** Writing – review and editing; funding acquisition. **Erick Forno:** Writing – review and editing; funding acquisition.

## FUNDING INFORMATION

This study was in part supported by NIH grants K01HL166436 (AHO), R03HS029088 (AHO), R01HL170368 (EF), and P01HL158507 (BG).

## CONFLICT OF INTEREST STATEMENT

None.

## Supporting information


Figure S1.

Table S1.
