# Supplementary material for: Prognostic utility of pre‐biologic treatment correlates of childhood severe asthma exacerbation risk: Real world evidence
Source: Pediatr Allergy Immunol. 2025 Dec 3;36(12):e70247. doi: 10.1111/pai.70247 (PMC12673514; doi:10.1111/pai.70247)
Supplement: Supplementary file 1 — Figure S1. [file PAI-36-e70247-s001.docx]

**Supplemental Figure 1. Study Design Schematic and Flow Chart**

1.
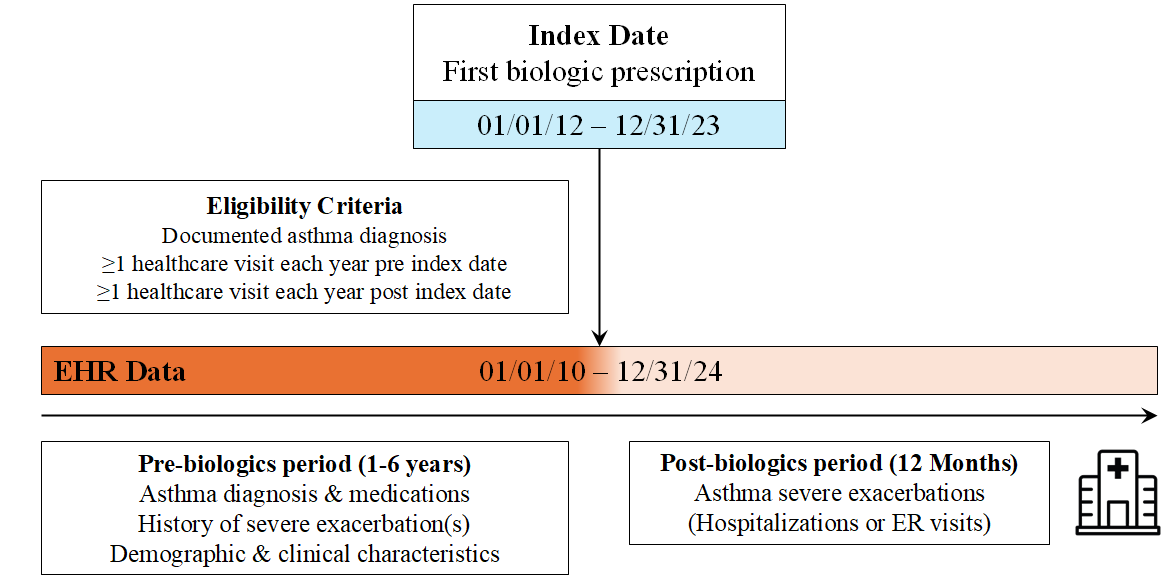
**Schematic design**
2. **Flowchart**

Children with asthma treated with a biologic by a pediatric pulmonologist or allergist at Riley / IUH 12/1/2019 and 12/31/2024

**N=166**

**Exclusion criteria**

1^st^ biologic date < asthma diagnosis date (n=33)

Age at 1^st^ biologic date < 6 years (n=11)

Analysis Sample

**n=122**

Encounter data accrued between January 1st, 2010, through December 31st, 2024, were examined to allow for a reasonable duration of patient follow-up period before and after biologic treatment initiation. This relatively long study period allowed us to include biologics approved for the treatment of children ≥6 years old between 2012 and 2023. The longer pre (vs post) biologic period was considered to ensure sufficient data was available to characterize early-childhood asthma risk factors (≤3 years of age), history of disease severity, and prior treatments.

**Supplemental Figure 2. Kaplan Meier plot of time to incident severe exacerbation 12-Months After the Biologic initiation**

| 1. **Overall** | 1. **PDM Risk** |
| --- | --- |
| 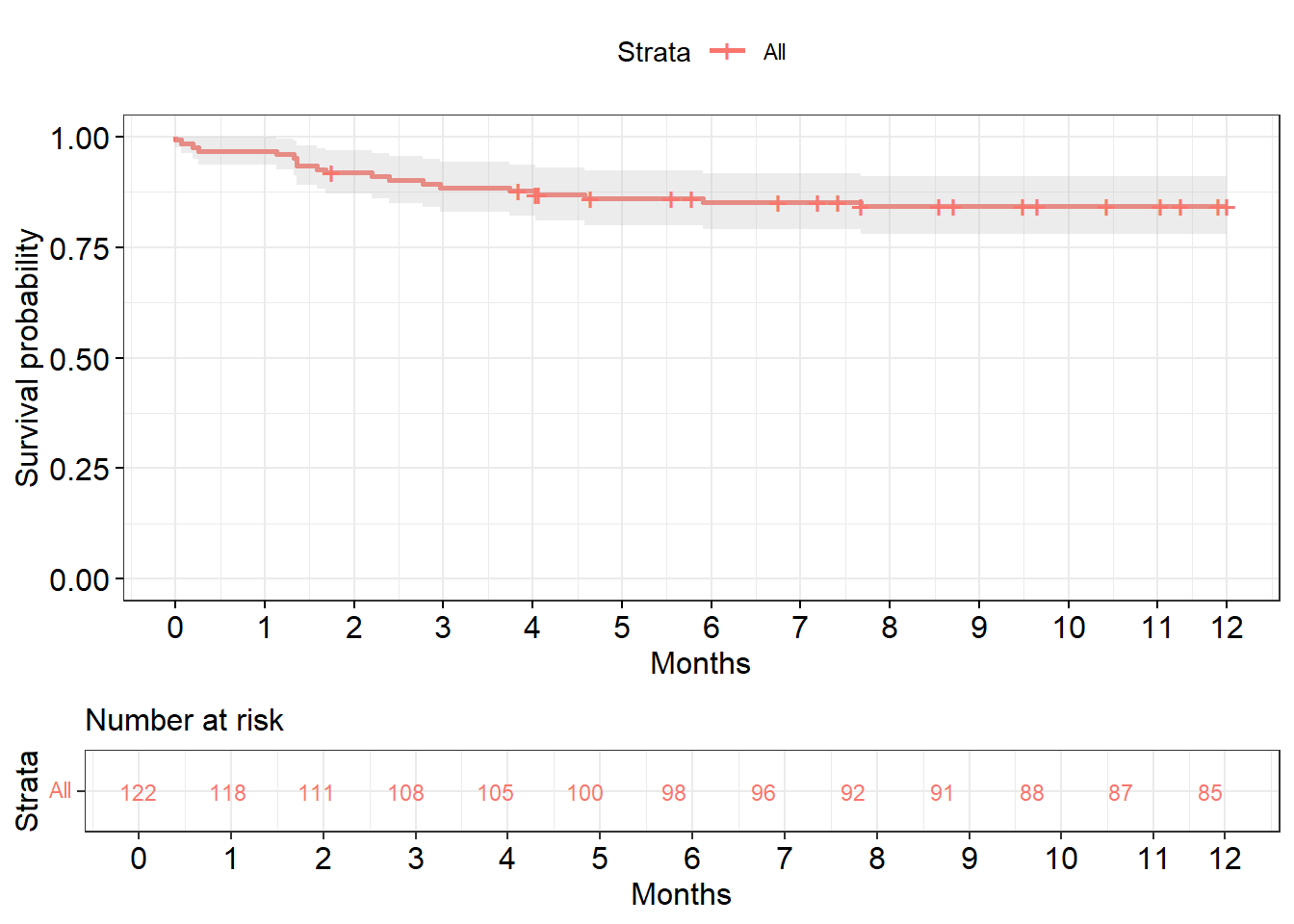 | 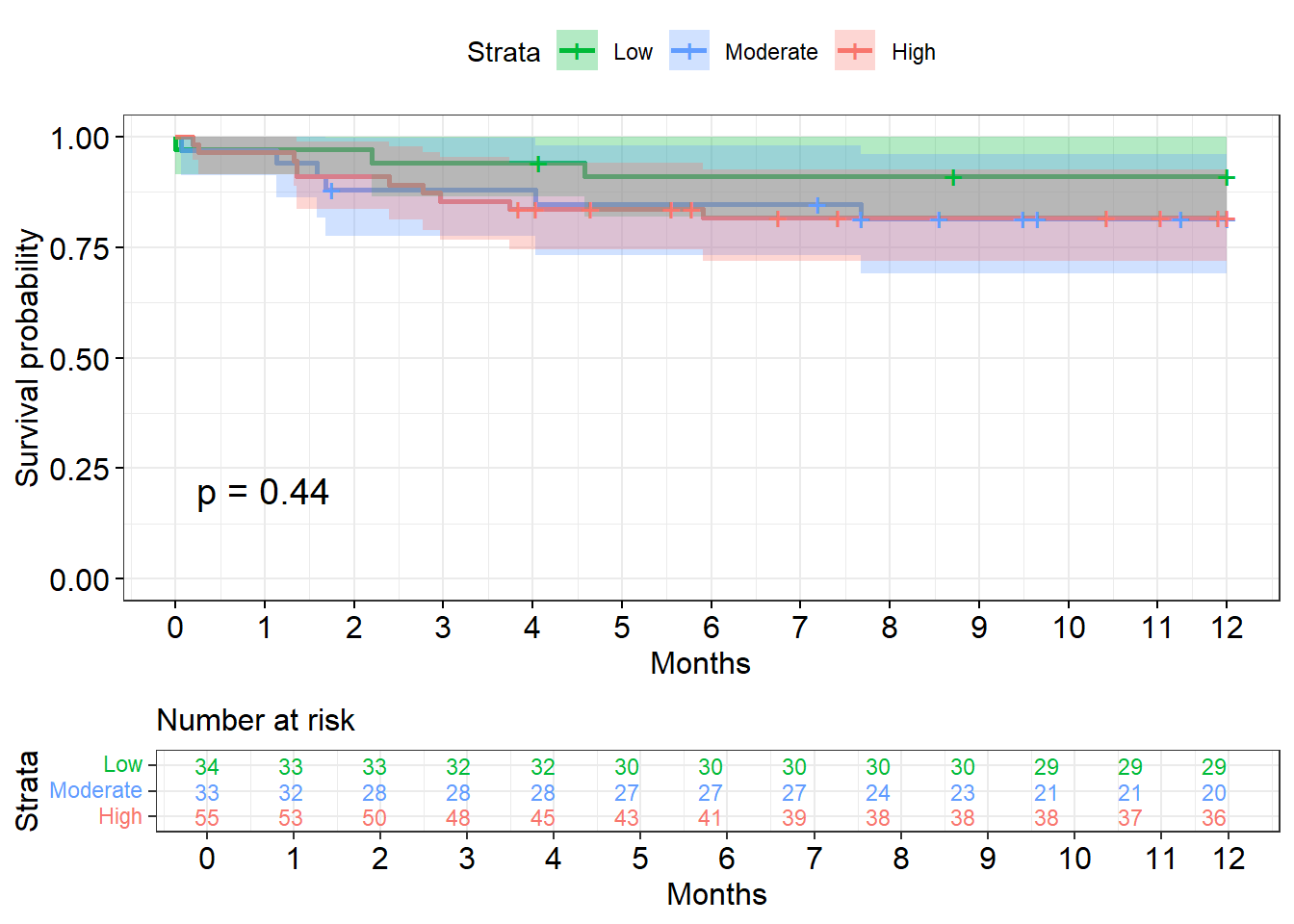 |
| 1. **SAE History (one year prior to biologic initiation)** | 1. **Biologic Type** |
| 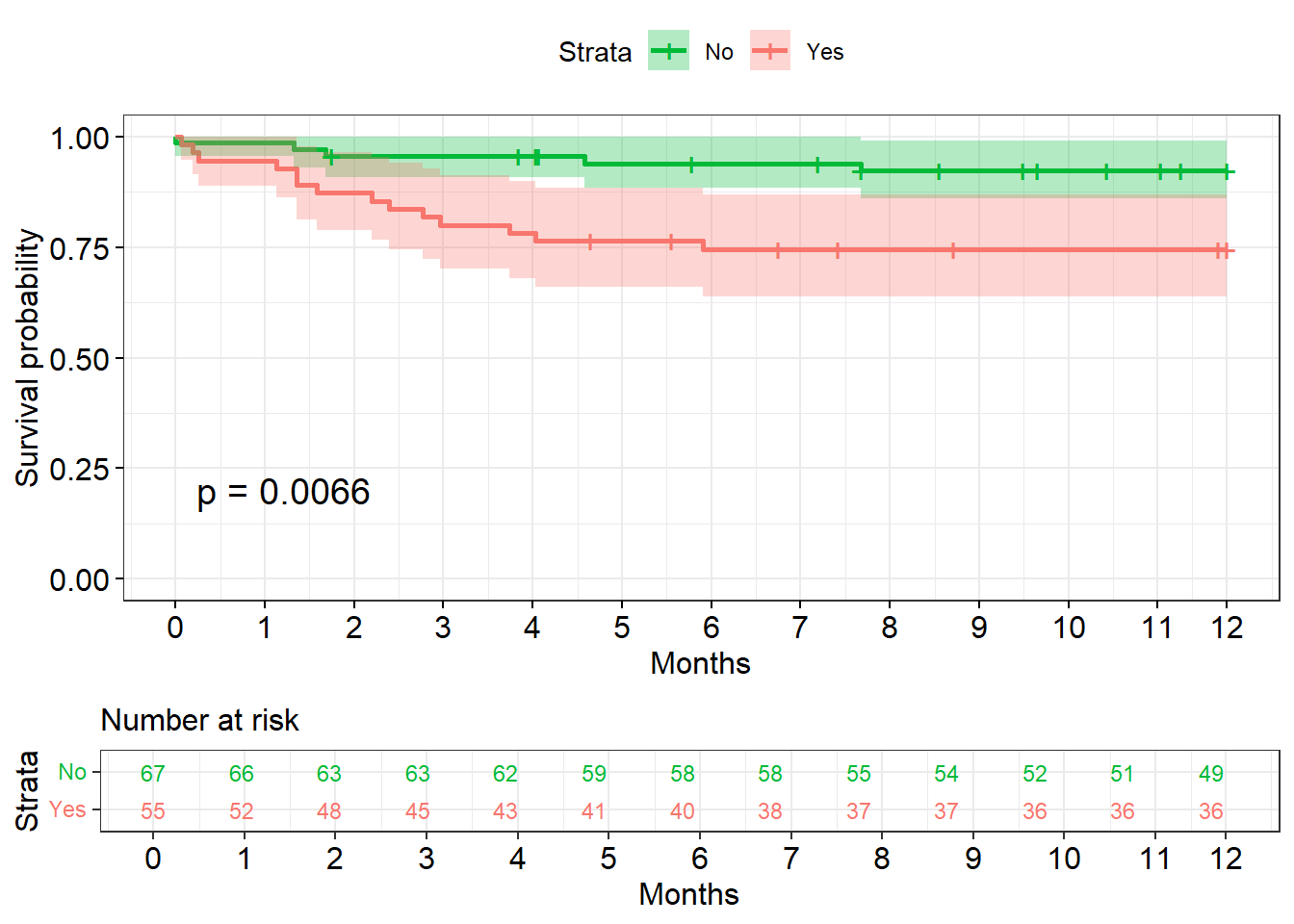 | 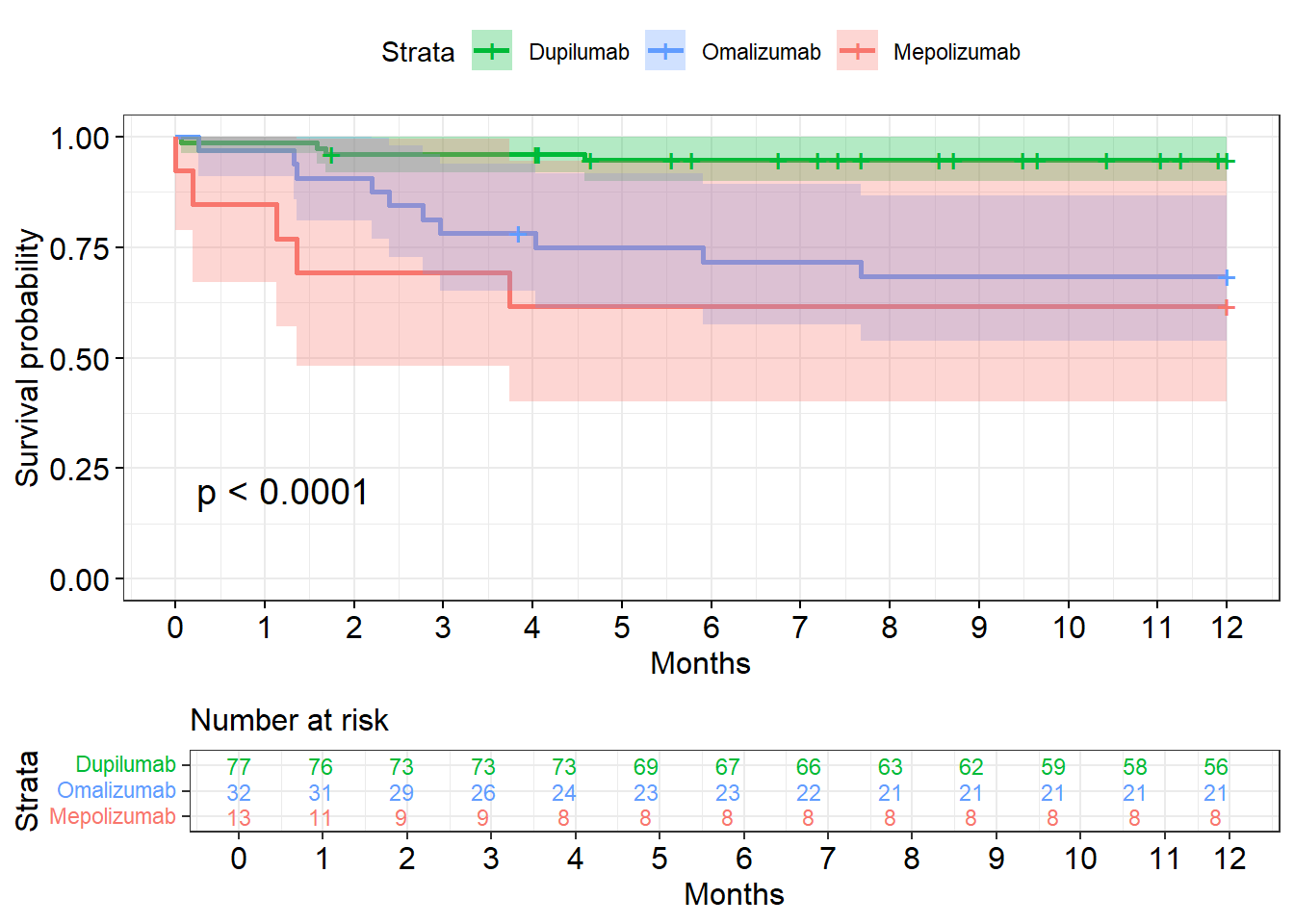 |

**Supplemental Figure 3: Correlation Matrix Between LASSO Predictors and SAE Risk Factors**


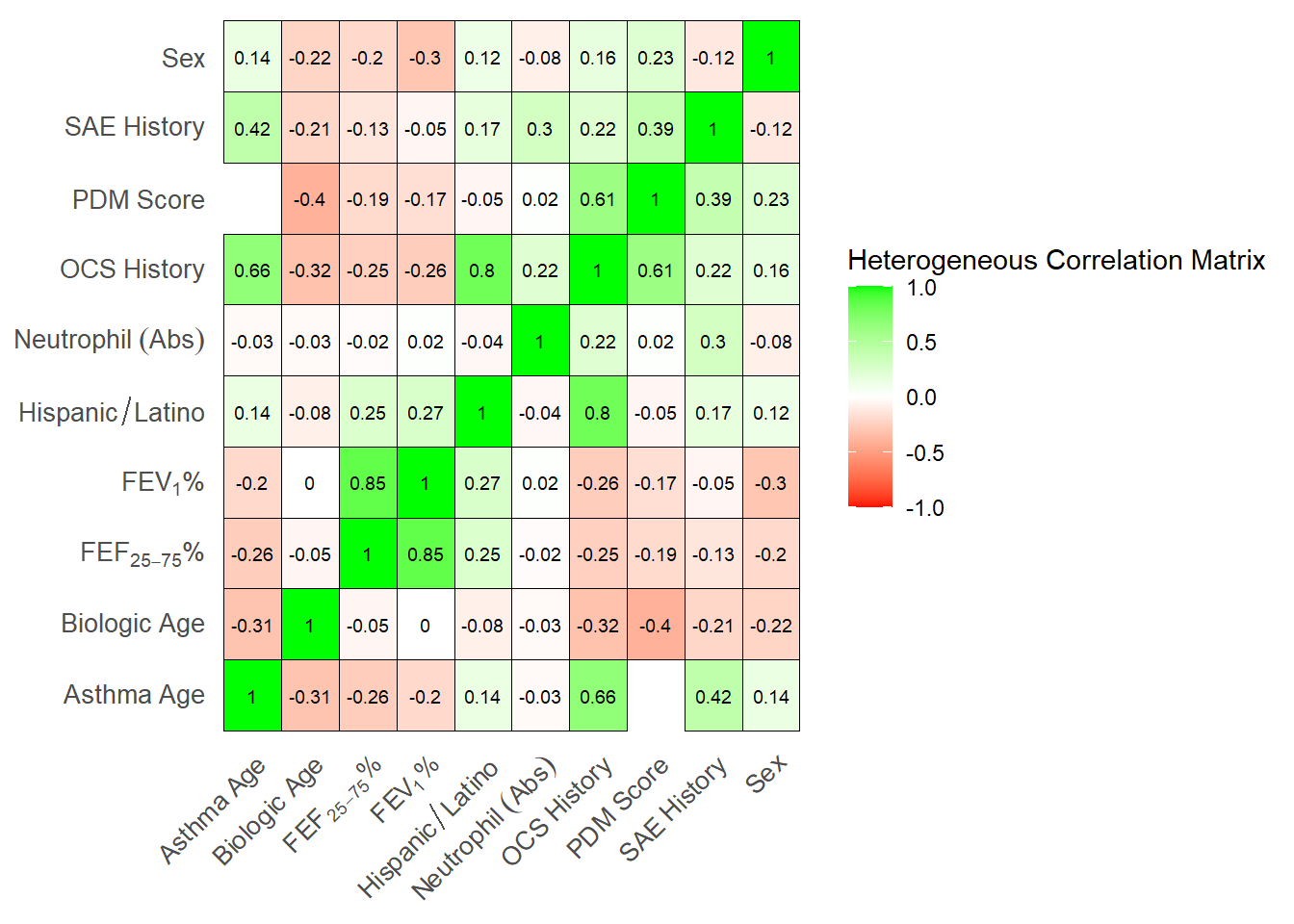


**Supplemental Figure 4: Prognostic performance of SAE prediction models (corrected for optimism) post-biologic initiation**

| 1. **ROC Curves** | 1. **Calibration Plots** |
| --- | --- |
| 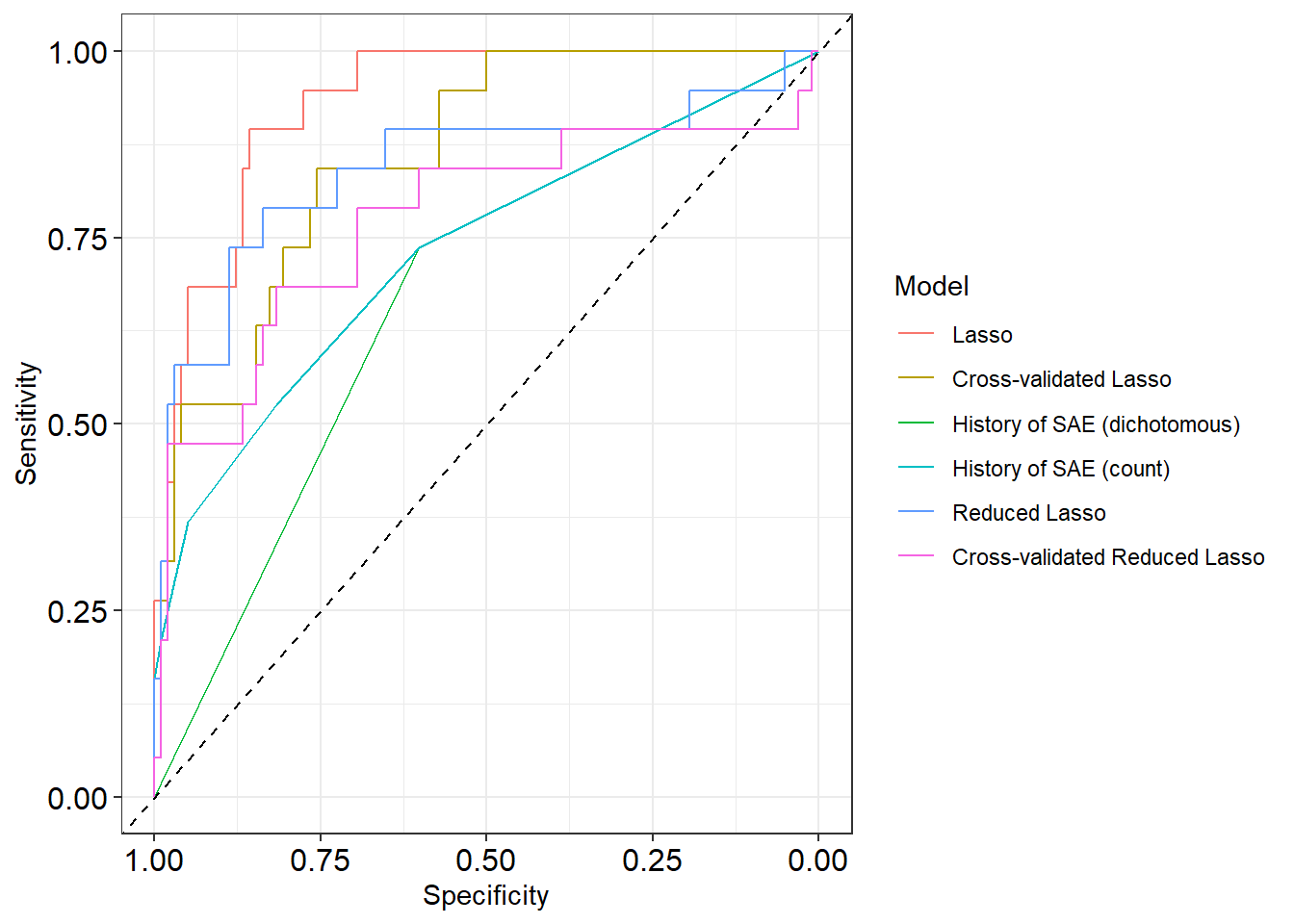 | 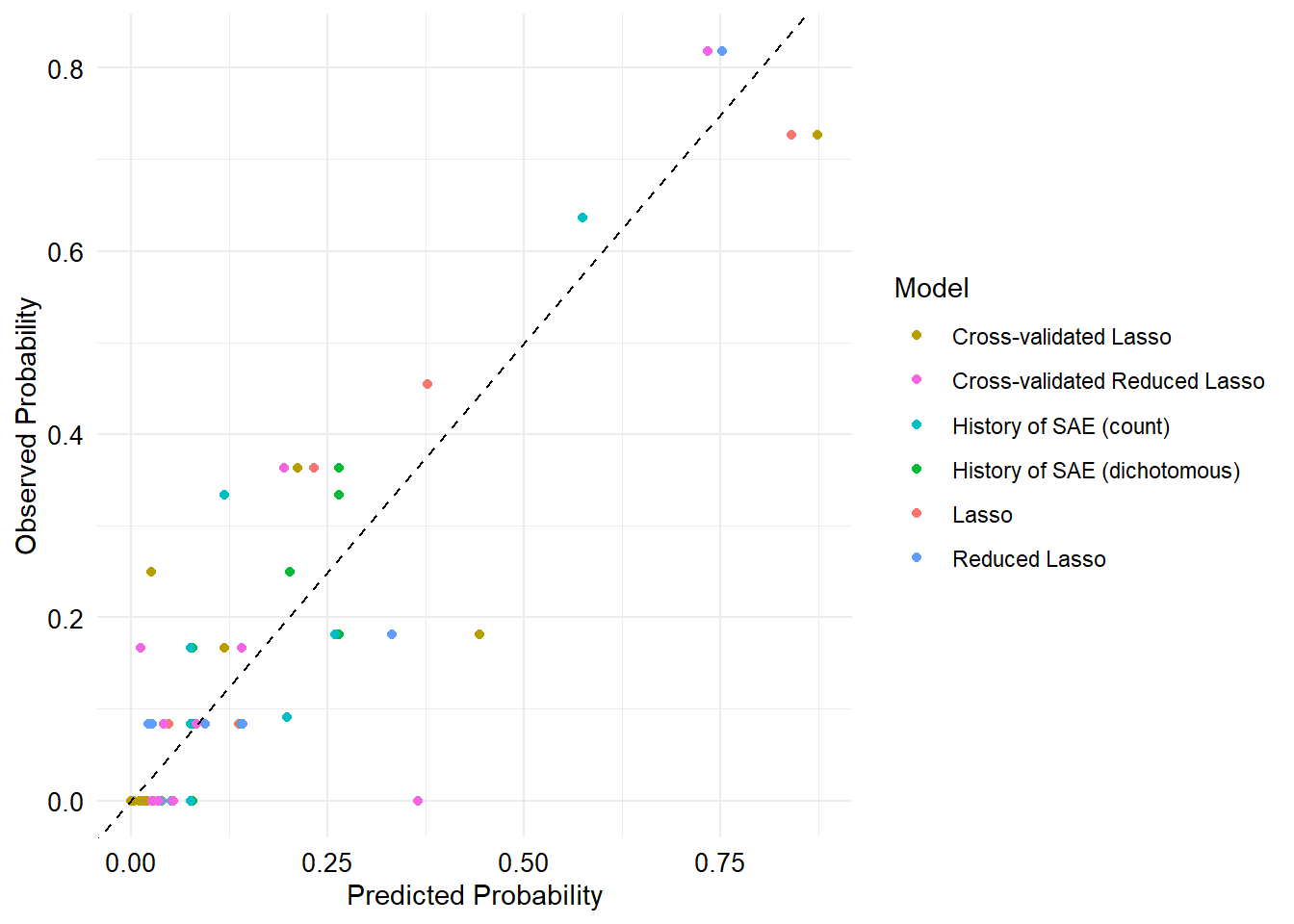 |
| 1. **Net-benefit Curves** | 1. **Lasso Model with a Cut-off point (≥0.20) for High Risk** |
| 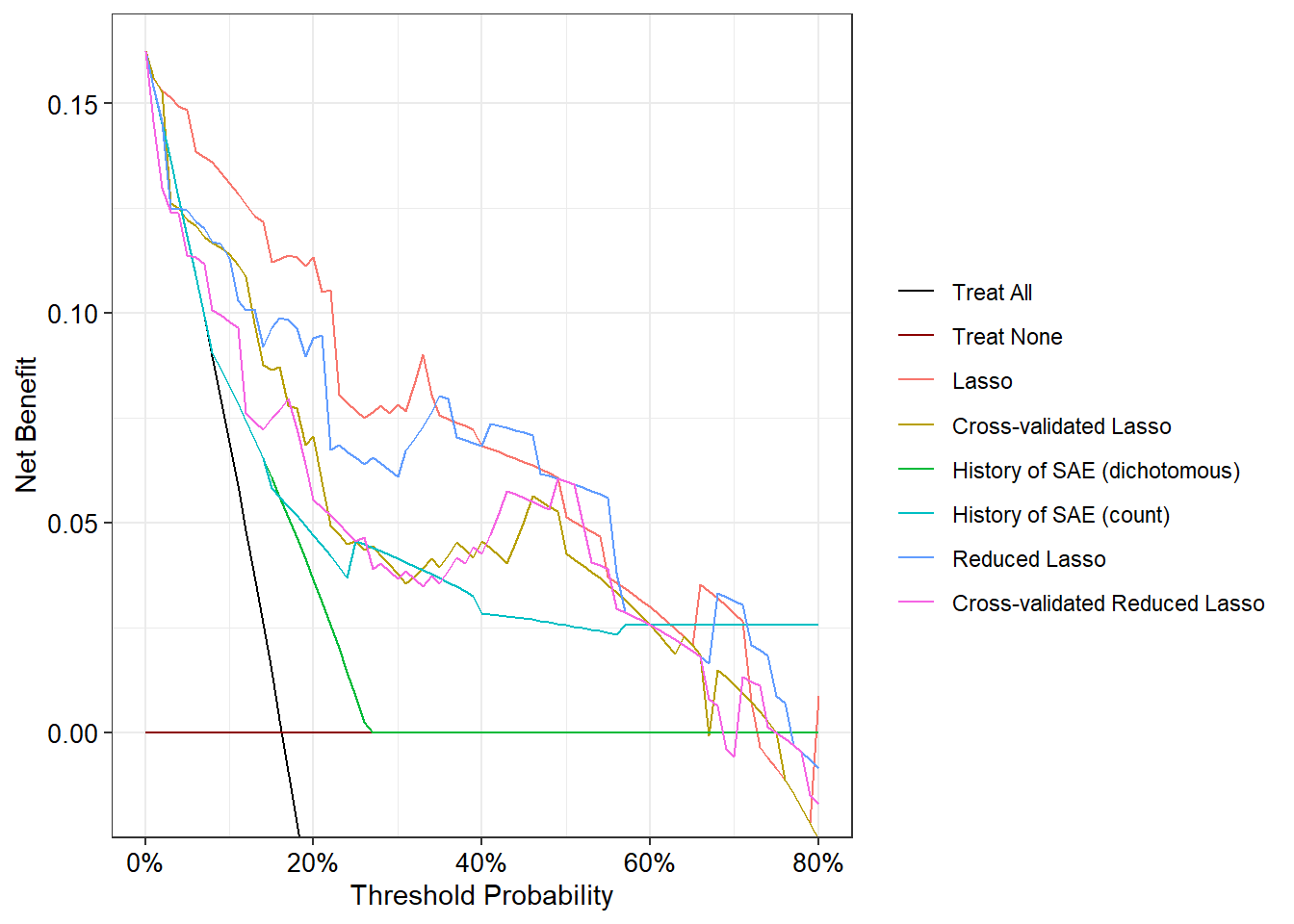 | 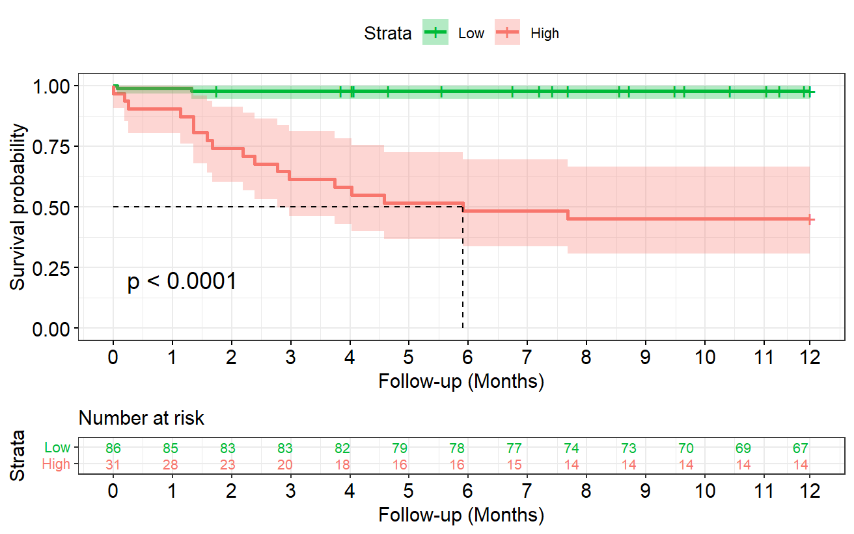 |

Comparison of the AUCs between the LASSO and crude Model (**A**); Comparison of Calibration plots between LASSO and crude model **(B**); Net-benefit comparisons across different risk threshold (**C**); Kaplan-Meir plot of the LASSO prognostic model risk strata based on a risk threshold of 0.2 for high-risk (**D**)

**Supplemental Figure 5: Nomogram for Lasso model**


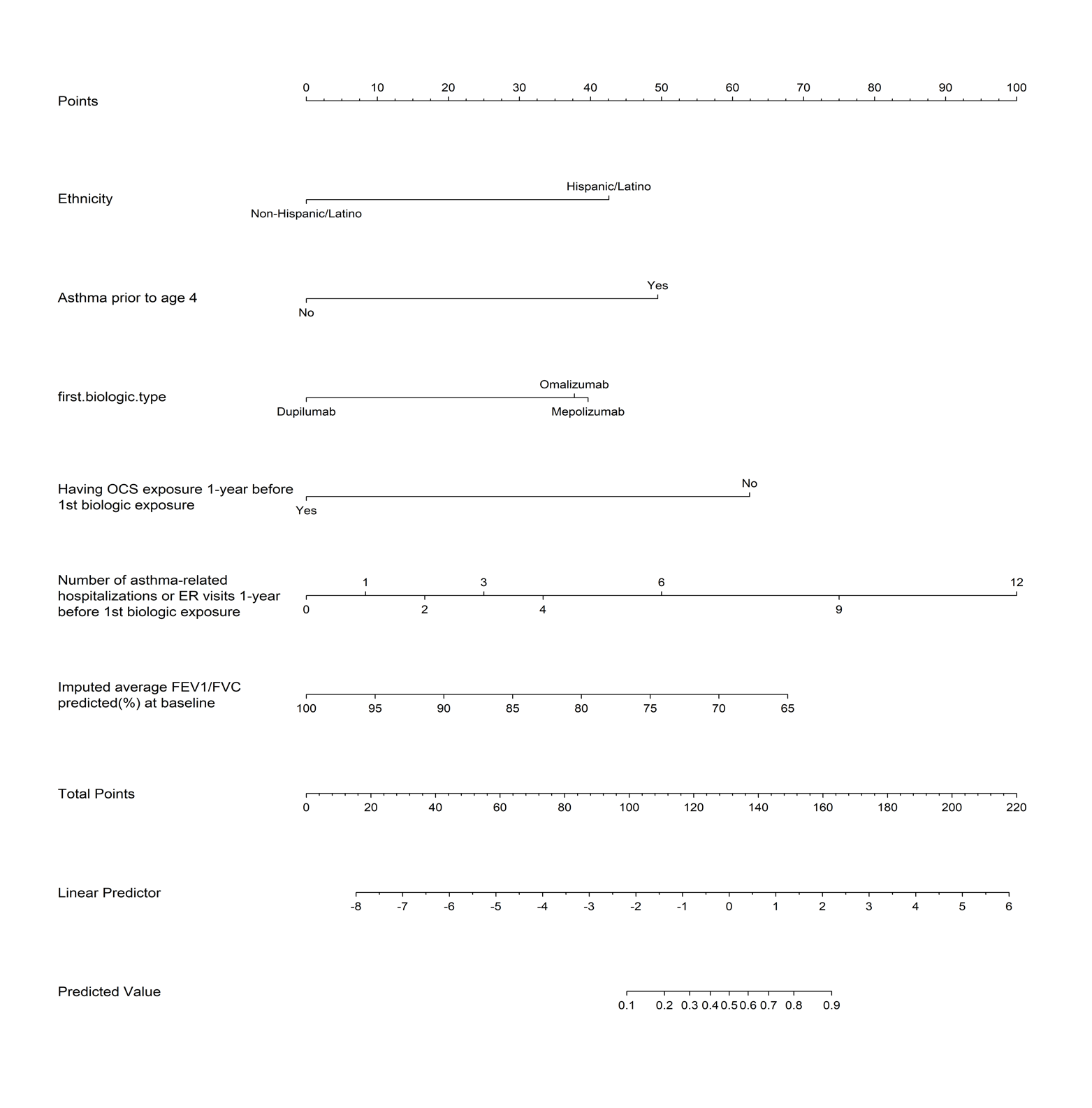


The nomogram can be used to aid in clinical decision-making. For instance, for patient who is Non-Hispanic/Latino (0 pts), with pre-school asthma diagnosis (50 pts), prescribed dupilumab (0 pts), with a history of OCS (0 pts) and 1 SAE (7.5 pts), and with FEV1/FVC 90% predicted (20 pts), the total score is 77.5 pts, with a predicted probability of 0.02 (Predicted Value) from the lasso model. Based on the Youden cut-off point (0.20), this patient is categorized as low risk. Maintaining all the factors except the biologic agent, if this patient were prescribed omalizumab (37.5 pts), the total score would be 115 pts, with a predicted probability of 0.25 (Predicted Value), which correspond to a high risk of future SAE.

**Supplemental Figure 6. Bootstrap (1000 resamples) Density Plot of the Lasso Prognostic Performance Metrics (including 95% Intervals)**

| **Youden’s Index** | **Accuracy** | **Sensitivity** | **Specificity** |
| --- | --- | --- | --- |
| 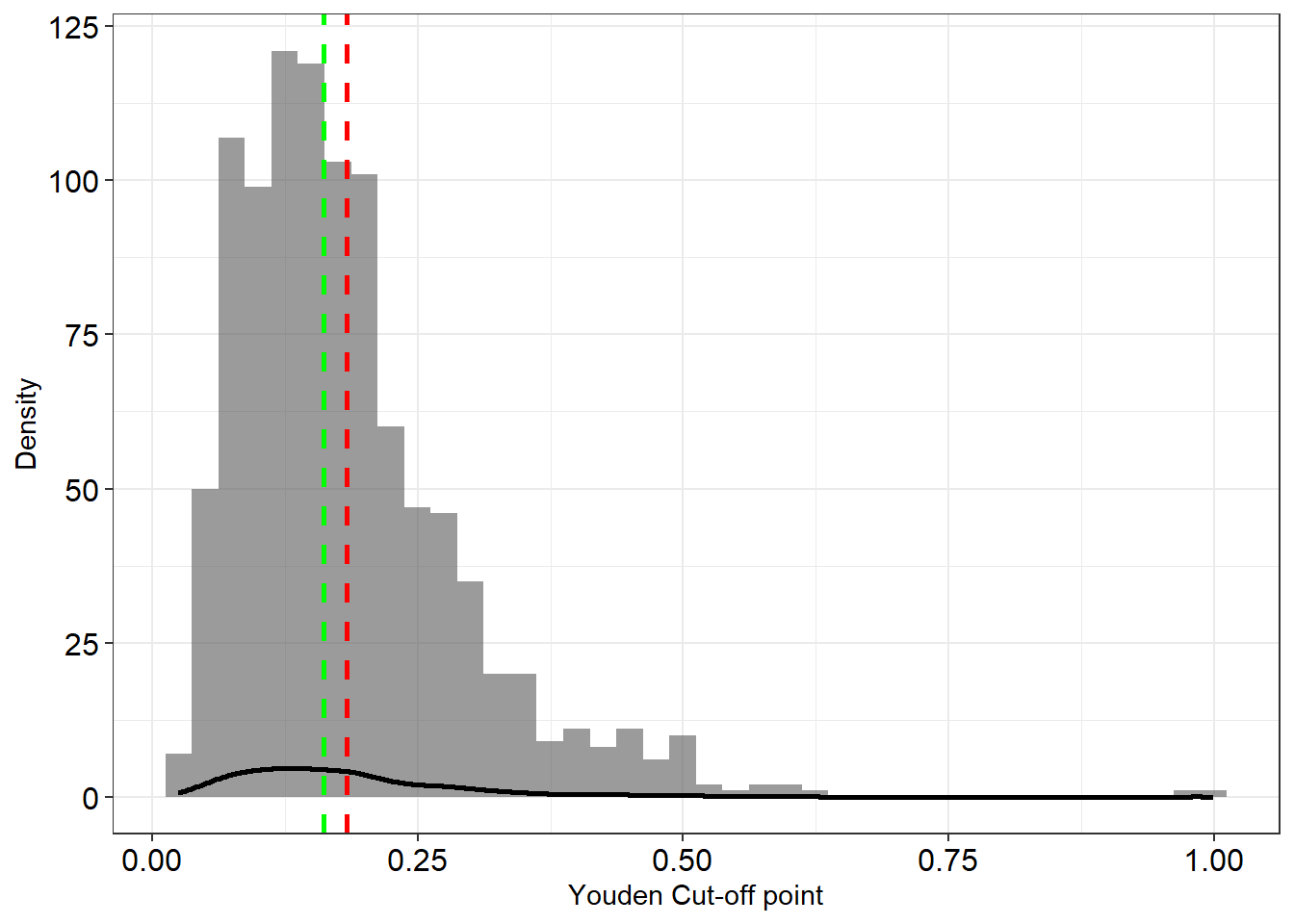  Mean: 0.18 (0.17, 0.19);  Median: 0.16 (0.16, 0.17) | 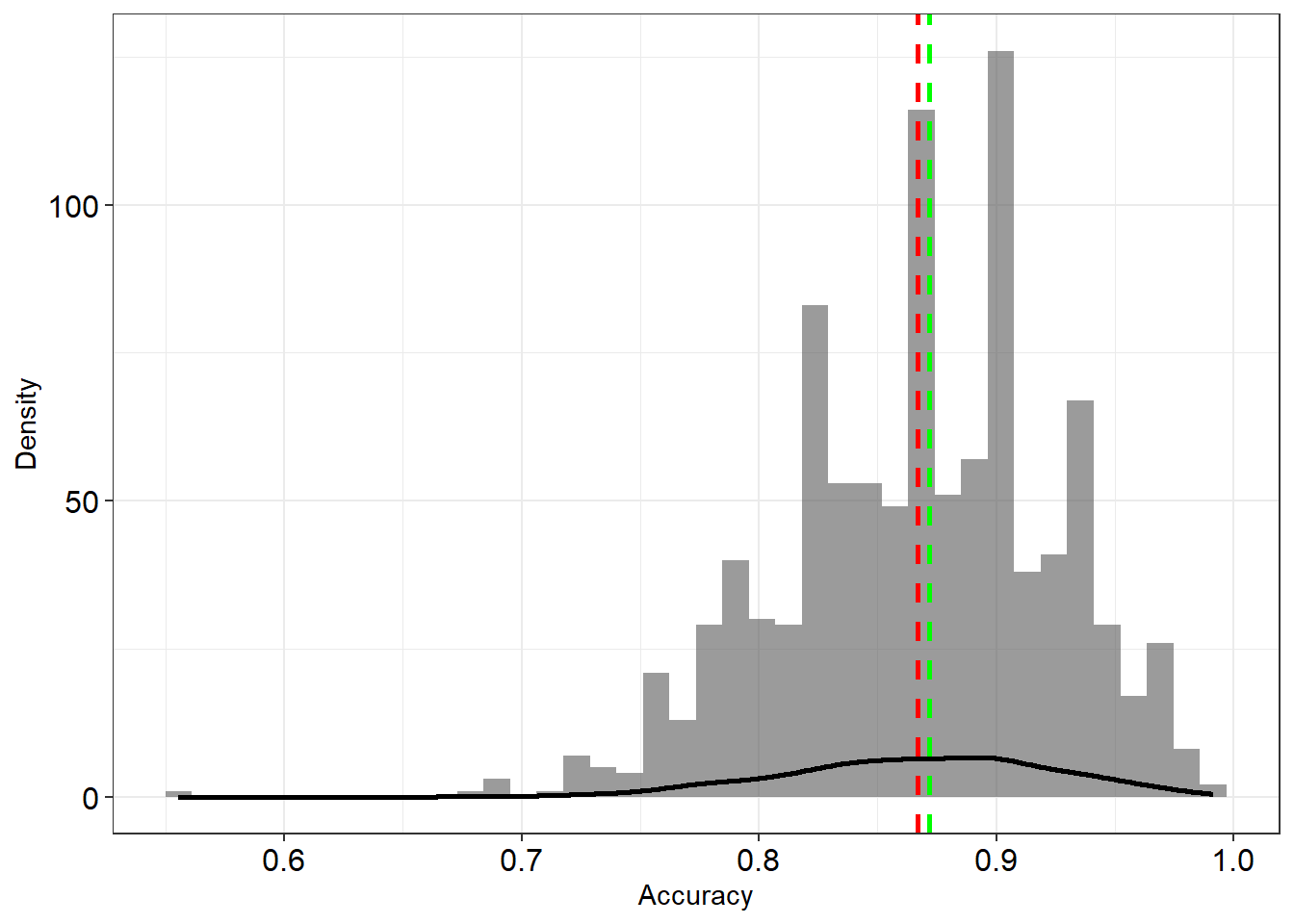  Mean: 0.87(0.86, 0.87);  Median: 0.87(0.86, 0.87) | 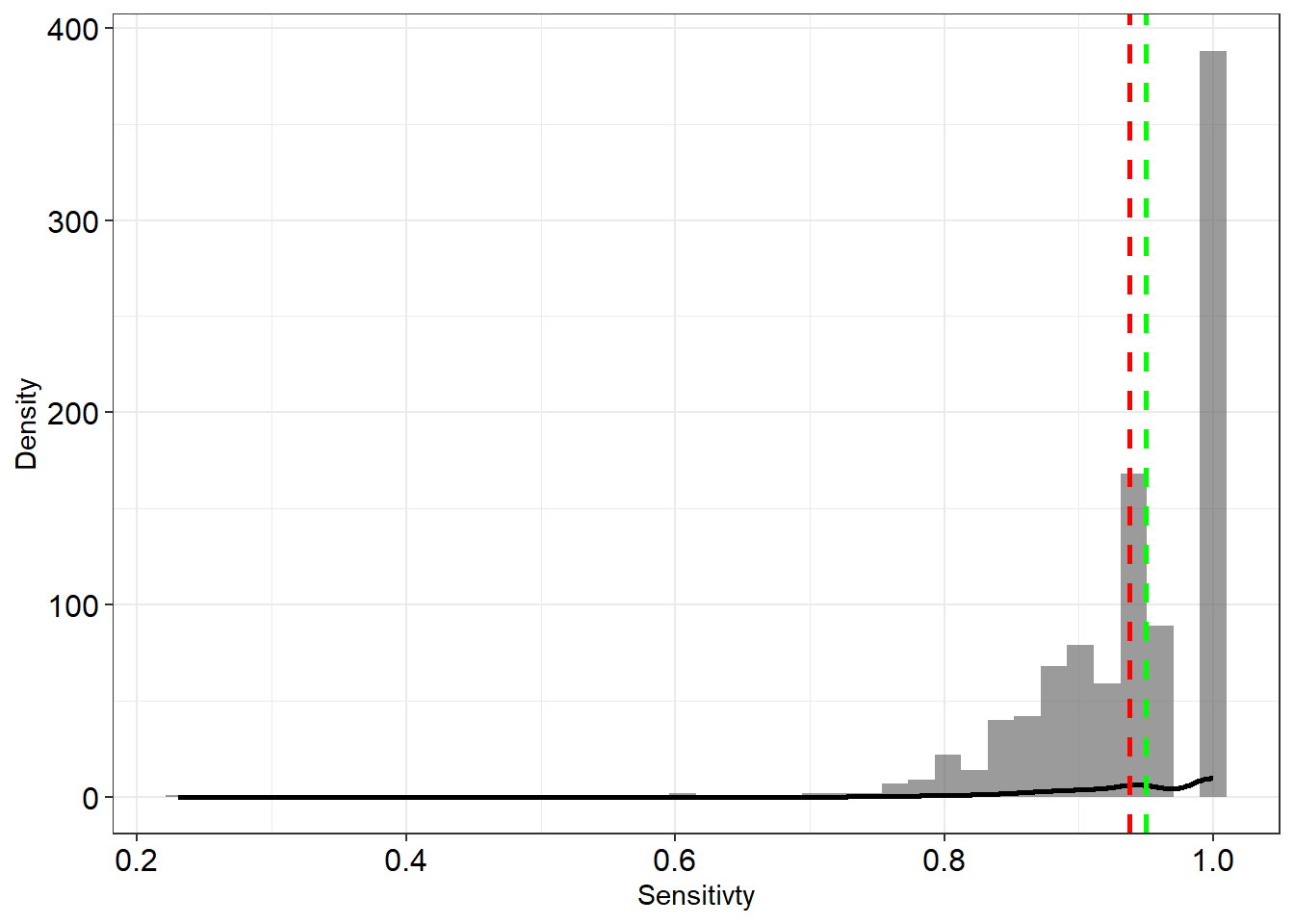  Mean: 0.93 (0.93, 0.94);  Median: 0.95 (0.94, 0.95) | 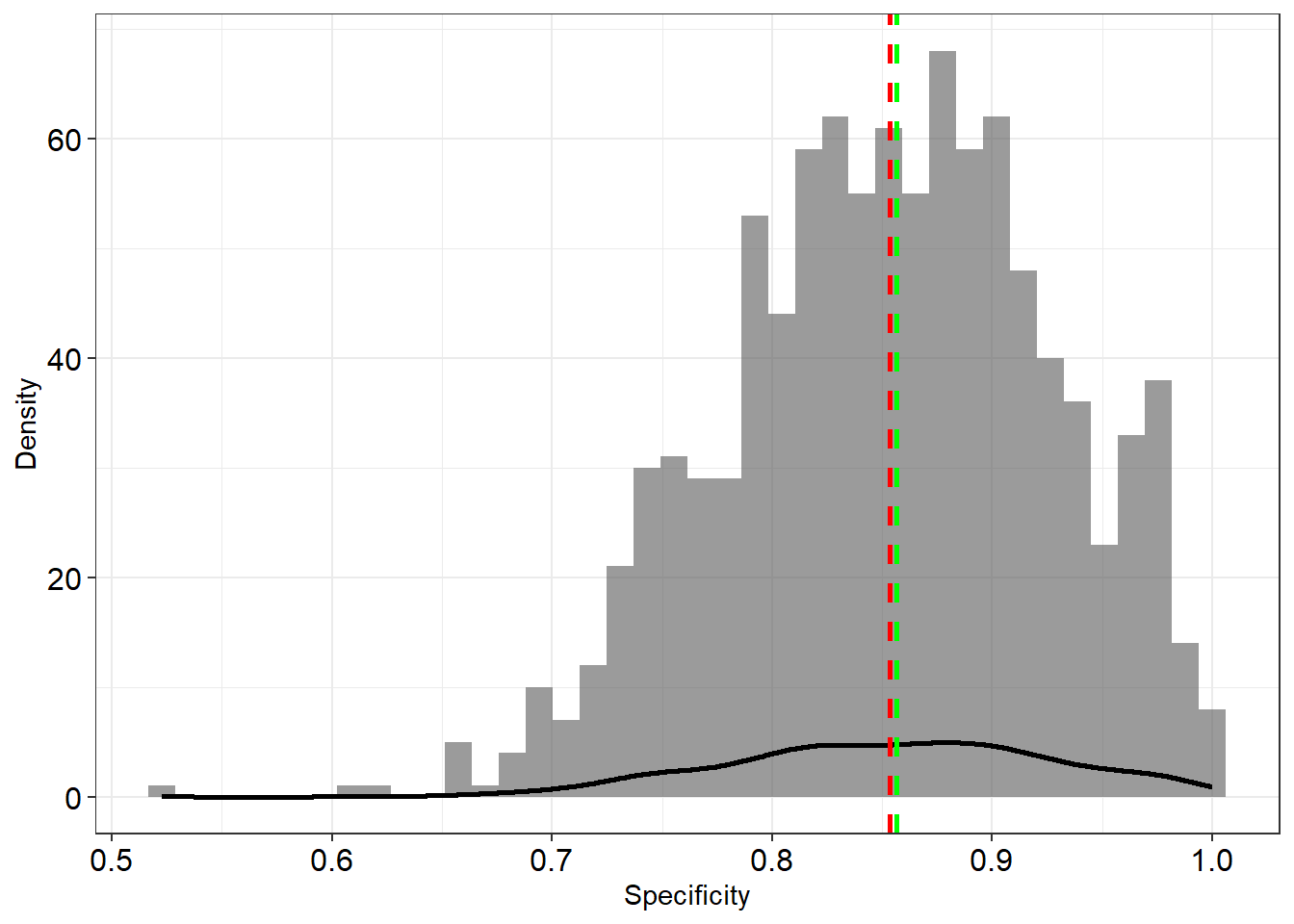  Mean: 0.85 (0.85, 0.86);  Median: 0.86 (0.85, 0.86) |
| **Positive Predictive Value** | **Negative Predictive Value** | **Positive Likelihood Ratio** | **Negative Likelihood Ratio** |
| 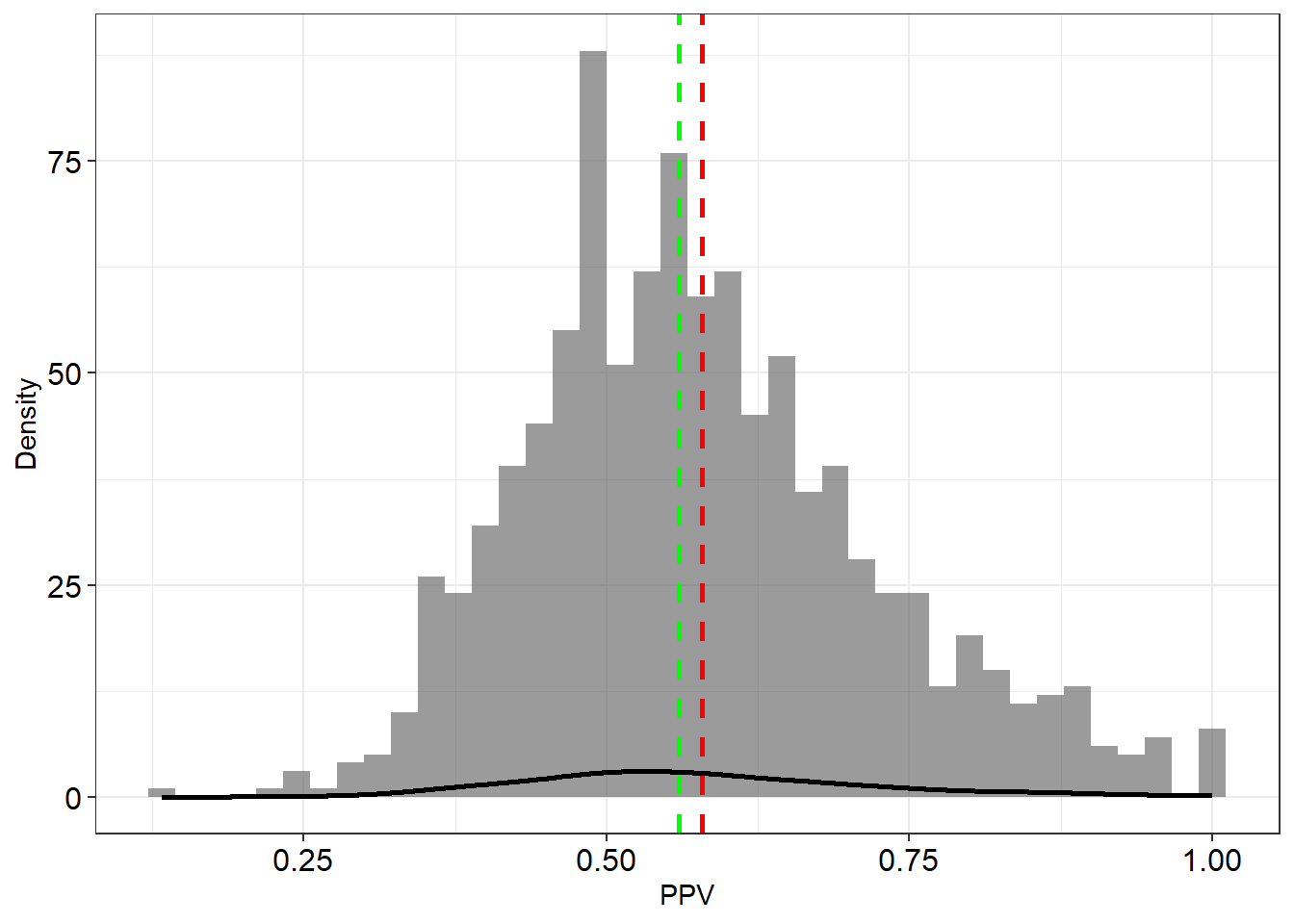  Mean: 0.57 (0.56, 0.59):  Median: 0.56 (0.55, 0.57) | 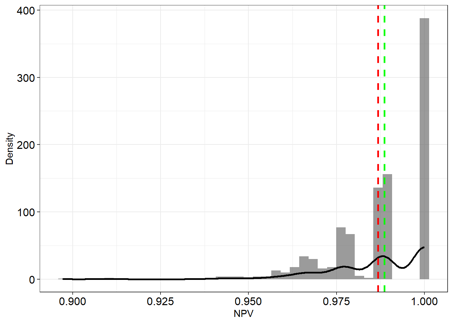  Mean: 0.98 (0.98, 0.98),  Median:0.98 (0.98, 0.98) | 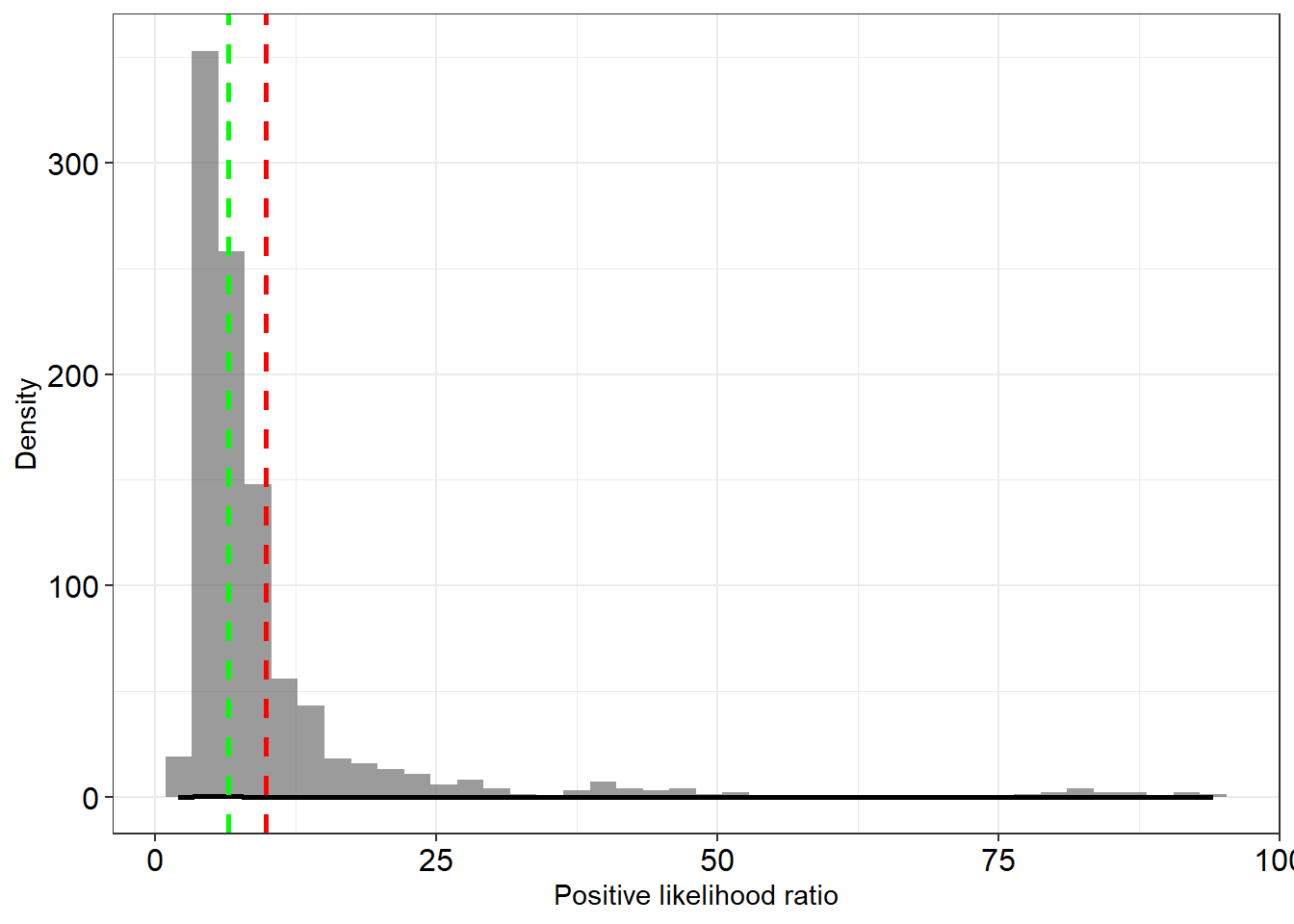  Mean: 9.89 (9.22, 10.68);  Median: 6.53 (6.26, 6.78) | 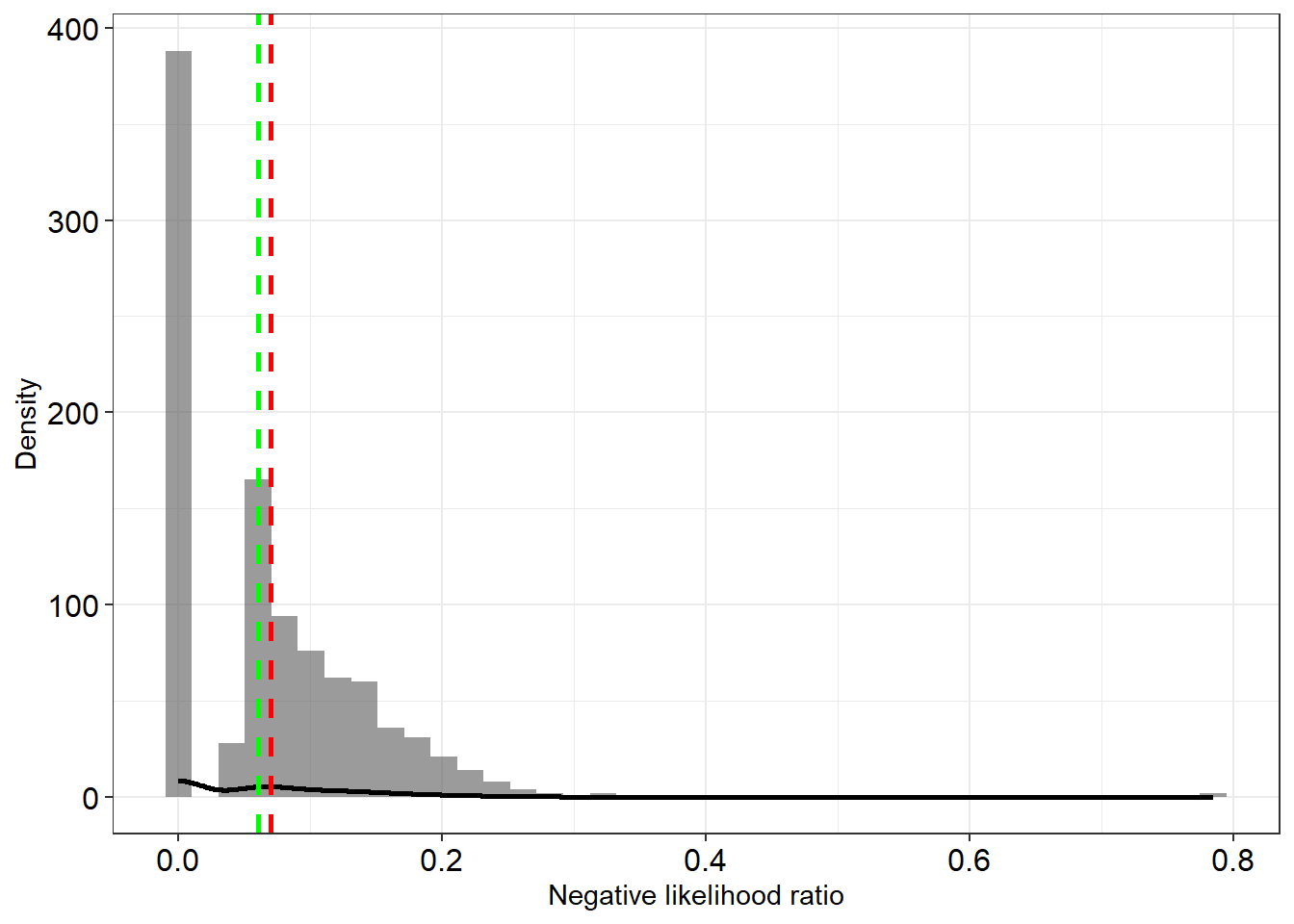  Mean: 0.07 (0.06, 0.07);  Median: 0.06 (0.05, 0.06) |
