# Supplementary material for: Prognostic utility of pre‐biologic treatment correlates of childhood severe asthma exacerbation risk: Real world evidence
Source: Pediatr Allergy Immunol. 2025 Dec 3;36(12):e70247. doi: 10.1111/pai.70247 (PMC12673514; doi:10.1111/pai.70247)
Supplement: Supplementary file 2 — Table S1. [file PAI-36-e70247-s002.docx]

**Prognostic utility of pre-biologic treatment correlates of childhood severe asthma exacerbation risk: real world evidence**

**Online Supplementary Data**

Arthur H Owora, MPH PhD,^a,b^ Bowen Jiang, MS,^a^ Yash Shah, MS,^a^ Benjamin Gaston, MD,^a^ Erick Forno, MD MPH^a^

*^a^Division of Pediatric Pulmonology, Allergy/Immunology and Sleep Medicine, Department of Pediatrics, Indiana University School of Medicine, Indiana, USA; ^b^Center for Biomedical Informatics, Regenstrief Institute, Indiana, USA*

**Corresponding author:** Arthur H. Owora, PhD

Dept of Pediatrics, Indiana University School of Medicine

Riley Hospital for Children

705 Riley Hospital Drive, Indianapolis, IN 46202

ahowora@iu.edu, 317-274-9109

**Supplemental Table 1. Definitions and descriptions of study variables**

| **Characteristics** | Early childhood predictors of asthma | |
| --- | --- | --- |
| **Key Terms** | Passive digital marker (PDM) | ICD Codes |
| Asthma (Outcome) | Physician documented asthma diagnosis based on icd9/10 code/text | J45.xx, 493.xx |
| Pre-school age asthma (PF) | Physician documented asthma diagnosis based on icd9/10 code/text at ages ≤3 years | J45.xx, 493.xx |
| Black or African American Race | Documented self-report |  |
| Parental History of Asthma | Documented self-report | Z82.5, V17.5 |
| Wheezing | Physician documented wheeze symptoms or diagnosis in the first 3 years of life | 786.07, J80, R09.2, J96.xx, 518.82, 518.81, 799.1, R06.2 |
| Wheezing without Cold | Physician documented wheeze symptoms /diagnosis that **did not** include cold related symptoms/diagnosis in the first 3 years of life | ICD codes used for colds: J00, J31.1, J06.0, J06.9, 465.9, 460 |
| Eczema | Physician documented diagnosis of Eczema in the first 3 years of life | 692.5, 692.6, L30.x, L20 |
| Polysensitization/  Multiple allergies† | Two or more physician documented allergy diagnosis including allergy related reactions related to aeroallergens or foods  OR  Two or more laboratory results of positive allergy sensitization related to aeroallergens or foods based on skin prick test identified as an allergen wheal was ≥3 mm greater than the saline control or a positive allergy blood test of specific immunoglobulin E (IgE) ≥0.35 kU/l.  OR  At least one allergy diagnosis and one positive allergy sensitization result related to aeroallergens or foods | Z91.0, 995.3, T78.40XA |
| HCPC Allergy clusters‡ | Created from the allergy reports and allergy sensitization results described above |  |
| Pneumonia | Physician documented of a pneumonia diagnosis in the first three years of life | 481, 482, 483, 484, 485, 486, 487.0, J12, J13, J14, J15, J16, J17, J18 |
| Bronchiolitis | Physician documented a bronchiolitis diagnosis in the first 3 years of life | 466.1, 466.11, 466.19, J21.0, J21.1, J21.8, J21.9 |

†Descriptors of allergies/polysensitization documented in the EHR: (1) Pollen allergies include allergy to elm, pollen; (2) Dust/Dust Mites allergies include allergy to dust, dust mites, and dander; (3) Nut allergies include allergy to almonds, cashews, pistachios, pecans, peanuts, tree nuts, walnuts, and nuts; (4) Egg allergies include allergy to eggs; (5) Seafood allergies include allergy to crab, lobster, shrimp, fish, shellfish, swordfish, tuna, scallops, and seafood; (6) Seasonal allergies include allergy to fall, winter, autumn, summer, and seasonal; (7) Milk allergies include allergy to milk, and dairy; (8) Fruit allergies include allergy to banana, coconut, pineapple, kiwi, raspberry, strawberry, watermelon, mango and fruits; (9) Pet allergies include allergy to cats, dogs, mice, rodents, pet dander, animal dander, and animals; (10) Drug allergies include allergy to ibuprofen, amoxicillin, Augmentin, Tylenol, erythromycin, antibiotics, penicillin, propylene glycol and drugs; (11) Grass allergies include allergy to grass, and hay; (12) Insects allergies include allergy to wasps, cockroaches, insect stings, bees, hornets and insects; (13) Smoke allergies include allergy to smoke; (14) Mold allergies include allergy to mold; (15) Fur allergies include allergy to fur; (16) Trees/Plants allergies include allergy to birch, oak, pigweed, ragweed, trees and plants; (17) Other-Food allergies include allergy to wheat, gluten, rice, pork, soy, sunflower seeds and foods; (18) Other-General allergies include allergy to adhesive bandages, adhesives, dyes, environment, latex, metals, narcotics, perfumes, and other.

‡HCPC is a robust method that combines three standard multivariate data analysis methods (Multiple correspondence analysis or principal component analysis for categorical variables, Hierarchical clustering, and K-means clustering) to produce statistical clusters based on patient attributes. An agglomerative algorithm was used to determine the optimal number of clusters that minimized the total intra-cluster variation (i.e., total within-cluster variation or total within-cluster sum of square). To assess convergent validity of the derived clusters, univariate tests (Analysis of variance/Kruskal-Wallis test or Chi-square/Fisher’s exact tests) were used to test for dependence between allergy categories, early childhood asthma risk factors, and derived cluster groups.

**Supplemental Table 2. Power and Sample Size Considerations for a survival (Cox proportional hazard model) and logistic prognostic model**

| **Cox proportional hazard model** | | | | | | | |
| --- | --- | --- | --- | --- | --- | --- | --- |
|  | Sample size | Shrinkage | Parameter | Cox-Snell R-squared | Maximum R-squared | Nagelkerke _R-squared | Events Per Predictor (EPP) |
|  |  |  |  |  |  |  |  |
| Criteria 1 | 110 | 0.900 | 2 | 0.15 | 0.592 | 0.253 | 8.66 |
| Criteria 2 | 62 | 0.835 | 2 | 0.15 | 0.592 | 0.253 | 4.88 |
| Criteria 3 | 110 | 0.900 | 2 | 0.15 | 0.592 | 0.253 | 8.66 |
| **Final** | 110 | 0.900 | 2 | 0.15 | 0.592 | 0.253 | 8.66 |
| **Binary Logistic Model (C-statistic = 0.8, prevalence = 0.15, Cox-Snell R-sq = 0.1505)** | | | | | | | |
| Criteria 1 | 110 | 0.900 | 2 | 0.1505 | 0.571 | 0.264 | 8.25 |
| Criteria 2 | 64 | 0.841 | 2 | 0.1505 | 0.571 | 0.264 | 4.80 |
| Criteria 3 | 196 | 0.900 | 2 | 0.1505 | 0.571 | 0.264 | 14.70 |
| **Final** | 196 | 0.900 | 2 | 0.1505 | 0.571 | 0.264 | 14.70 |

Cox-Snell R-squared - proportion of variance explained. Prognostic model assumptions: 1) 0.05 acceptable difference in apparent & adjusted R-squared (Criteria 1: minimize overfitting), 2) 0.05 margin of error in estimation of overall risk at a 1-year time point (Criteria 2: minimize the absolute difference between the apparent and adjusted Nagelkerke's R-squared) and 3) Events per Predictor Parameter (EPP) assumes overall event rate of 0.15 (Criteria 3: precise estimation of the average outcome risk)

**Supplemental Table 3. Baseline characteristics by biologic type (N=122)**

| Characteristic | Dupilumab N = 77 | Omalizumab N = 32 | Mepolizumab N = 13 | P-value |
| --- | --- | --- | --- | --- |
| Age of 1^st^ asthma (years) |  |  |  | 0.328 |
| Mean (SD) | 3.3 (3.7) | 2.6 (3.7) | 3.4 (3.9) |  |
| Age of 1^st^ Biologic (years) |  |  |  | 0.065 |
| Mean (SD) | 11.4 (3.5) | 10.0 (2.7) | 11.8 (2.4) |  |
| Sex |  |  |  | 0.637 |
| Female | 37 (48.1%) | 17 (53.1%) | 8 (61.5%) |  |
| Male | 40 (51.9%) | 15 (46.9%) | 5 (38.5%) |  |
| Race |  |  |  | 0.470 |
| White | 30 (39.0%) | 8 (25.0%) | 5 (38.5%) |  |
| Black/African American | 44 (57.1%) | 23 (71.9%) | 7 (53.8%) |  |
| Others | 3 (3.9%) | 1 (3.1%) | 1 (7.7%) |  |
| PDM Score |  |  |  | 0.145 |
| Mean (SD) | 61 (36) | 74 (35) | 60 (36) |  |
| PDM risk |  |  |  | 0.422 |
| Low | 23 (29.9%) | 7 (21.9%) | 4 (30.8%) |  |
| Moderate | 24 (31.2%) | 6 (18.8%) | 3 (23.1%) |  |
| High | 30 (39.0%) | 19 (59.4%) | 6 (46.2%) |  |
| Absolute eosinophil at baseline |  |  |  | 0.212 |
| Mean (SD) | 0.47 (0.26) | 0.53 (0.41) | 0.64 (0.30) |  |
| Absolute neutrophil at baseline |  |  |  | 0.073 |
| Mean (SD) | 4.67 (2.06) | 5.43 (2.30) | 6.04 (3.43) |  |
| ACT score at baseline |  |  |  | 0.102 |
| Mean (SD) | 17.0 (4.1) | 16.7 (2.9) | 14.9 (3.2) |  |
| Eosinophil (%) at baseline |  |  |  | 0.286 |
| Mean (SD) | 5.8 (3.4) | 6.0 (4.6) | 7.8 (4.7) |  |
| FEF _25-75_ predicted (%) at baseline |  |  |  | 0.338 |
| Mean (SD) | 68 (27) | 63 (23) | 55 (24) |  |
| FEV_1_/FVC predicted (%) at baseline |  |  |  | 0.303 |
| Mean (SD) | 88.8 (1.86) | 88.5 (5.07) | 88.2 (1.82) |  |
| Average FEV1 predicted (%) at baseline |  |  |  | 0.526 |
| Mean (SD) | 89 (19) | 89 (16) | 83 (21) |  |
| Neutrophil **(**%) at baseline |  |  |  | 0.148 |
| Mean (SD) | 53 (12) | 58 (15) | 56 (14) |  |
| Total IgE (log-transformed) |  |  |  | 0.582 |
| Mean (SD) | 6.29 (1.48) | 6.42 (1.23) | 6.54 (1.34) |  |

**Supplemental Table 4. Distribution of patient characteristics and medication history by incidence of at least one severe exacerbation (SE) 12-month post-biologic**

| **Patient Characteristics** | **Overall** | **No SAE** | **SAE** | p value | Odds Ratio (95%CI) | |
| --- | --- | --- | --- | --- | --- | --- |
| **Overall** | **122** | 103 (84.4%) | 19 (15.6%) |  | Crude | Adjusted |
| **Sex** |  |  |  | 0.502 |  |  |
| Female | 62 (50.8%) | 51 (82.3%) | 11 (17.7%) |  | — | — |
| Male | 60 (49.2%) | 52 (86.7%) | 8 (13.3%) |  | 0.70 (0.25, 1.87) | 0.57 (0.20, 1.56) |
| **Race** |  |  |  | 0.239 |  |  |
| White | 43 (35.2%) | 39 (90.7%) | 4 (9.3%) |  | — | — |
| Black/African American | 74 (60.7%) | 59 (79.7%) | 15 (20.3%) |  | 2.48 (0.83, 9.19) | 2.10 (0.66, 8.15) |
| Others | 5 (4.1%) | 5 (100.0%) | 0 (0.0%) |  | — | — |
| **Ethnicity** |  |  |  | 0.172 |  |  |
| Non-Hispanic/Latino | 117 (95.9%) | 100 (85.5%) | 17 (14.5%) |  | — | — |
| Hispanic/Latino | 5 (4.1%) | 3 (60.0%) | 2 (40.0%) |  | 3.73 (0.47, 24.1) | 3.35 (0.40, 23.0) |
| **PDM Score** |  |  |  | 0.093 |  |  |
| Mean (SD) | 64 (36) | 62 (36) | 76 (33) |  | — | — |
| Median (Q1, Q3) | 73 (27, 94) | 68 (23, 93) | 91 (46, 99) |  | 1.01 (1.00, 1.03) | 1.00 (0.98, 1.02) |
| **PDM risk** |  |  |  | 0.442 |  |  |
| Low | 34 (27.9%) | 31 (91.2%) | 3 (8.8%) |  | — | — |
| Moderate | 33 (27.0%) | 27 (81.8%) | 6 (18.2%) |  | 2.23 (0.53, 11.4) | 0.28 (0.02, 4.49) |
| High | 55 (45.1%) | 45 (81.8%) | 10 (18.2%) |  | 2.25 (0.62, 10.7) | 0.09 (0.00, 6.06) |
| **Age in years,** Median (Q1, Q3) |  |  |  |  |  |  |
| Asthma Diagnosis | 2.0 (1.0, 4.0) | 2.0 (1.0, 5.0) | 1.0 (0.0, 1.0) | 0.006 | **0.83 (0.64, 0.99)** | 0.83 (0.59, 1.07) |
| Biologic initiation | 11.0 (8.0, 13.0) | 11.0 (8.0, 13.0) | 10.0 (8.0, 14.0) | 0.419 | 0.94 (0.81, 1.10) | 0.99 (0.83, 1.16) |
| **Allergies** |  |  |  | 0.718 |  |  |
| No | 66 (54.1%) | 55 (83.3%) | 11 (16.7%) |  | — | — |
| Yes | 56 (45.9%) | 48 (85.7%) | 8 (14.3%) |  | 0.82 (0.30, 2.21) | 0.39 (0.10, 1.40) |
| **Eczema** |  |  |  | 0.479 |  |  |
| No | 54 (44.3%) | 47 (87.0%) | 7 (13.0%) |  | — | — |
| Yes | 68 (55.7%) | 56 (82.4%) | 12 (17.6%) |  | 1.46 (0.54, 4.20) | 0.94 (0.29, 3.23) |
| **Early Childhood Wheezing (≤3 yrs)** |  |  |  | 0.406 |  |  |
| No | 49 (40.2%) | 43 (87.8%) | 6 (12.2%) |  | — | — |
| Yes | 73 (59.8%) | 60 (82.2%) | 13 (17.8%) |  | 1.49 (0.54, 4.55) | 0.37 (0.04, 2.99) |
| **Wheezing without a cold (≤3 years)** |  |  |  | 0.364 |  |  |
| No | 50 (41.0%) | 44 (88.0%) | 6 (12.0%) |  | — | — |
| Yes | 72 (59.0%) | 59 (81.9%) | 13 (18.1%) |  | 1.56 (0.57, 4.75) | 0.45 (0.05, 3.74) |
| **Parental asthma** |  |  |  | 0.167 |  |  |
| No | 69 (56.6%) | 61 (88.4%) | 8 (11.6%) |  | — | — |
| Yes | 53 (43.4%) | 42 (79.2%) | 11 (20.8%) |  | 2.08 (0.77, 5.82) | 2.14 (0.72, 6.62) |
| **Bronchiolitis** |  |  |  | 0.620 |  |  |
| No | 83 (68.0%) | 71 (85.5%) | 12 (14.5%) |  | — | — |
| Yes | 39 (32.0%) | 32 (82.1%) | 7 (17.9%) |  | 1.26 (0.43, 3.45) | 0.79 (0.24, 2.50) |
| **Pneumonia** |  |  |  | 0.325 |  |  |
| No | 64 (52.5%) | 56 (87.5%) | 8 (12.5%) |  | — | — |
| Yes | 58 (47.5%) | 47 (81.0%) | 11 (19.0%) |  | 1.55 (0.58, 4.33) | 1.08 (0.32, 3.87) |
| **Pre-school asthma** |  |  |  | 0.041 |  |  |
| No | 37 (30.3%) | 35 (94.6%) | 2 (5.4%) |  | — | — |
| Yes | 85 (69.7%) | 68 (80.0%) | 17 (20.0%) |  | **4.32 (1.14, 28.2)** | 3.90 (0.27, 83.1) |
| **Biologic agent** |  |  |  | <0.001 |  |  |
| Dupilumab | 77 (63.1%) | 73 (94.8%) | 4 (5.2%) |  | — | — |
| Omalizumab | 32 (26.2%) | 22 (68.8%) | 10 (31.3%) |  | **8.33 (2.51, 33.0)** | **6.70 (1.71, 30.9)** |
| Mepolizumab | 12 (9.8%) | 7 (58.3%) | 5 (41.7%) |  | **12.5 (2.74, 62.2)** | **8.89 (1.48, 55.1)** |
| **Cluster by Allergen Test** |  |  |  | 0.179 | — | — |
| 1 | 79 (64.8%) | 70 (88.6%) | 9 (11.4%) |  | 2.70 (0.86, 8.24) | 2.70 (0.82, 8.77) |
| 2 | 27 (22.1%) | 20 (74.1%) | 7 (25.9%) |  | 1.69 (0.34, 6.61) | 2.17 (0.41, 9.48) |
| 3 | 16 (13.1%) | 13 (81.3%) | 3 (18.8%) |  |  |  |
| **ICS 1-year before biologic initiation** |  |  |  | 0.716 |  |  |
| No | 43 (35.2%) | 37 (86.0%) | 6 (14.0%) |  | — | — |
| Yes | 79 (64.8%) | 66 (83.5%) | 13 (16.5%) |  | 1.26 (0.45, 3.84) | 1.30 (0.45, 4.11) |
| **ICS+LABA 1-year before biologics** |  |  |  | 0.694 |  |  |
| No | 14 (11.5%) | 13 (92.9%) | 1 (7.1%) |  | — | — |
| Yes | 108 (88.5%) | 90 (83.3%) | 18 (16.7%) |  | 2.51 (0.45, 47.2) | 1.91 (0.30, 37.3) |
| **OCS 1-year before biologics** |  |  |  | 0.230 |  |  |
| No | 14 (11.5%) | 10 (71.4%) | 4 (28.6%) |  | — | — |
| Yes | 108 (88.5%) | 93 (86.1%) | 15 (13.9%) |  | 0.38 (0.11, 1.54) | **0.06 (0.00, 0.41)** |
|  |  |  |  |  |  |  |
| **SAE 1-year before biologics** |  |  |  | 0.006 |  |  |
| No | 67 (54.9%) | 62 (92.5%) | 5 (7.5%) |  | — | — |
| Yes | 55 (45.1%) | 41 (74.5%) | 14 (25.5%) |  | **4.24 (1.49, 14.0)** | **3.49 (1.17, 12.0)** |
| **SAEs 1-year before biologics** |  |  |  | <0.001 |  |  |
| Mean (SD) | 0.94 (1.67) | 0.63 (0.92) | 2.63 (3.24) |  | **2.00 (1.38, 3.14)** | **1.89 (1.27, 3.13)** |
| **BMI within 1-yr of biologics** |  |  |  | 0.711 |  |  |
| Mean (SD) | 24 (8) | 24 (8) | 24 (8) |  | 0.99 (0.93, 1.05) | 1.02 (0.94, 1.09) |
| **BMI within 1-yr of biologics** |  |  |  | 0.956 |  |  |
| Underweight | 37 (30.3%) | 31 (83.8%) | 6 (16.2%) |  | — | — |
| Healthy Weight | 40 (32.8%) | 34 (85.0%) | 6 (15.0%) |  | 0.91 (0.26, 3.20) | 1.00 (0.27, 3.79) |
| Overweight | 18 (14.8%) | 16 (88.9%) | 2 (11.1%) |  | 0.60 (0.08, 2.99) | 0.90 (0.11, 5.31) |
| Obesity | 27 (22.1%) | 22 (81.5%) | 5 (18.5%) |  | 1.15 (0.30, 4.32) | 2.03 (0,40, 10.8) |
| **OCS 1-month before biologics** |  |  |  | 0.150 |  |  |
| No | 57 (46.7%) | 51 (89.5%) | 6 (10.5%) |  | — | — |
| Yes | 65 (53.3%) | 52 (80.0%) | 13 (20.0%) |  | 2.17 (0.79, 6.59) | 1.99 (0.70, 6.19) |
| **SABA 1-month before biologics** |  |  |  | 0.462 |  |  |
| No | 15 (12.3%) | 14 (93.3%) | 1 (6.7%) |  | — | — |
| Yes | 107 (87.7%) | 89 (83.2%) | 18 (16.8%) |  | 3.00 (0.55, 56.1) | 2.61 (0.45, 49.6) |
| **Absolute eosinophil at baseline** |  |  |  | 0.997 |  |  |
| Mean (SD) | 0.50 (0.32) | 0.50 (0.28) | 0.56 (0.47) |  | — | — |
| Median (Q1, Q3) | 0.50 (0.30, 0.62) | 0.50 (0.30, 0.62) | 0.53 (0.30, 0.67) |  | 1.86 (0.40, 7.87) | 1.75 (0.39, 7.63) |
| **Absolute neutrophil at baseline** |  |  |  | 0.024 |  |  |
| Mean (SD) | 5.02 (2.32) | 4.80 (2.17) | 6.17 (2.81) |  | **1.24 (1.02, 1.51)** | **1.23 (1.01, 1.51)** |
| **ACT score at baseline** |  |  |  | 0.197 |  |  |
| Mean (SD) | 16.7 (3.8) | 16.8 (4.0) | 15.9 (2.1) |  | 0.93 (0.81, 1.06) | 0.92 (0.80, 1.06) |
| **Eosinophil (%) at baseline** |  |  |  | 0.711 |  |  |
| Mean (SD) | 6.1 (3.9) | 6.1 (3.8) | 5.9 (4.5) |  | 0.99 (0.87, 1.12) | 0.99 (0.86, 1.11) |
| **FEF _25-75_ predicted (%)** |  |  |  | 0.309 |  |  |
| Mean (SD) | 65 (26) | 67 (27) | 59 (18) |  | 0.99 (0.97, 1.01) | 0.99 (0.97, 1.01) |
| **FEV_1_/FVC predicted (%)** |  |  |  | 0.646 |  |  |
| Mean (SD) | 88.65 (3.02) | 88.92 (1.99) | 87.18 (6.02) |  | 1.00 (0.97, 1.02) | 0.88 (0.68, 1.03) |
| **FEV_1_ predicted (%) at baseline** |  |  |  | 0.662 |  |  |
| Mean (SD) | 88 (18) | 88 (19) | 88 (15) |  | 1.86 (0.40, 7.87) | 1.00 (0.98, 1.03) |
| **Neutrophil (%) at baseline** |  |  |  | 0.133 |  |  |
| Mean (SD) | 55 (13) | 54 (13) | 60 (15) |  | 1.03 (0.99, 1.07) | 1.02 (0.99, 1.06) |
| **(log) Total IgE** |  |  |  | 0.829 |  |  |
| Mean (SD) | 6.35 (1.40) | 6.36 (1.39) | 6.28 (1.48) |  | 0.96 (0.68, 1.36) | 0.93 (0.65, 1.33) |

Adjusted models include PDM score, biologic agent, number of SAEs in the year prior to first biologic exposure, and baseline predicted FEV_1_/FVC (%)

**Supplemental Table 5. Association between patient characteristics and time to post-biologic SAE summarized by hazard ratios**

| **Patient Characteristics** | **Overall** | **No SAE** | **SAE** | p value † | Hazard Ratio (95%CI) | | |
| --- | --- | --- | --- | --- | --- | --- | --- |
| **Overall** | **122** | 103 (84.4%) | 19 (15.6%) |  | Crude | Adjusted |  |
| **Sex** |  |  |  | 0.440 |  |  |  |
| Female | 62 (50.8%) | 51 (82.3%) | 11 (17.7%) |  | — | — |  |
| Male | 60 (49.2%) | 52 (86.7%) | 8 (13.3%) |  | 0.70 (0.28, 1.74) | 0.60 (0.24, 1.49) |  |
| **Race** |  |  |  | 0.142 |  |  |  |
| White | 43 (35.2%) | 39 (90.7%) | 4 (9.3%) |  | — | — |  |
| Black/African American | 74 (60.7%) | 59 (79.7%) | 15 (20.3%) |  | 2.23 (0.74, 6.73) | 1.86 (0.59, 5.92) |  |
| Others | 5 (4.1%) | 5 (100.0%) | 0 (0.0%) |  | — | — |  |
| **Ethnicity** |  |  |  | 0.093 |  |  |  |
| Non-Hispanic/Latino | 117 (95.9%) | 100 (85.5%) | 17 (14.5%) |  | — | — |  |
| Hispanic/Latino | 5 (4.1%) | 3 (60.0%) | 2 (40.0%) |  | 3.27 (0.75, 14.2) | 3.24 (0.72, 14.6) |  |
| **PDM Score** |  |  |  |  |  |  |  |
| Mean (SD) | 64 (36) | 62 (36) | 76 (33) |  | 1.01 (1.00, 1.02) | 1.00 (0.97, 1.02) |  |
| **PDM risk** |  |  |  | 0.468 |  |  |  |
| Low | 34 (27.9%) | 31 (91.2%) | 3 (8.8%) |  | — | — |  |
| Moderate | 33 (27.0%) | 27 (81.8%) | 6 (18.2%) |  | 2.12 (0.53, 8.49) | 0.39 (0.03, 4.67) |  |
| High | 55 (45.1%) | 45 (81.8%) | 10 (18.2%) |  | 2.14 (0.59, 7.77) | 0.17 (0.00, 7.03) |  |
| Mean (SD)**Age in years** |  |  |  |  |  |  |  |
| Asthma Diagnosis | 3.1 (3.7) | 3.4 (3.8) | 1.7 (3.1) |  | 0.84 (0.68, 1.03) | 0.83 (0.63, 1.11) |  |
| Biologic initiation | 11.1 (3.3) | 11.2 (3.3) | 10.6 (3.3) |  | 0.95 (0.82, 1.09) | 0.98 (0.84, 1.15) |  |
| **Allergies** |  |  |  | 0.732 |  |  |  |
| No | 66 (54.1%) | 55 (83.3%) | 11 (16.7%) |  | — | — |  |
| Yes | 56 (45.9%) | 48 (85.7%) | 8 (14.3%) |  | 0.85 (0.34, 2.12) | 0.47 (0.15, 1.45) |  |
| **Eczema** |  |  |  | 0.499 |  |  |  |
| No | 54 (44.3%) | 47 (87.0%) | 7 (13.0%) |  | — | — |  |
| Yes | 68 (55.7%) | 56 (82.4%) | 12 (17.6%) |  | 1.38 (0.54, 3.50) | 0.91 (0.31, 2.70) |  |
| **Early Childhood Wheezing(≤3years)** |  |  |  | 0.469 |  |  |  |
| No | 49 (40.2%) | 43 (87.8%) | 6 (12.2%) |  | — | — |  |
| Yes | 73 (59.8%) | 60 (82.2%) | 13 (17.8%) |  | 1.43 (0.54, 3.76) | 0.42 (0.07, 2.62) |  |
| **Wheezing without a cold (≤3 years)** |  |  |  | 0.421 |  |  |  |
| No | 50 (41.0%) | 44 (88.0%) | 6 (12.0%) |  | — | — |  |
| Yes | 72 (59.0%) | 59 (81.9%) | 13 (18.1%) |  | 1.48 (0.56, 3.91) | 0.51 (0.08, 3.19) |  |
| **Parental asthma** |  |  |  | 0.132 |  |  |  |
| No | 69 (56.6%) | 61 (88.4%) | 8 (11.6%) |  | — | — |  |
| Yes | 53 (43.4%) | 42 (79.2%) | 11 (20.8%) |  | 1.99 (0.80, 4.94) | 2.01 (0.75, 5.40) |  |
| **Bronchiolitis** |  |  |  | 0.637 |  |  |  |
| No | 83 (68.0%) | 71 (85.5%) | 12 (14.5%) |  | — | — |  |
| Yes | 39 (32.0%) | 32 (82.1%) | 7 (17.9%) |  | 1.25 (0.49, 3.18) | 0.84 (0.30, 2.35) |  |
| **Pneumonia** |  |  |  | 0.375 |  |  |  |
| No | 64 (52.5%) | 56 (87.5%) | 8 (12.5%) |  | — | — |  |
| Yes | 58 (47.5%) | 47 (81.0%) | 11 (19.0%) |  | 1.51 (0.61, 3.75) | 1.10 (0.36, 3.39) |  |
| **Pre-school asthma** |  |  |  | 0.054 |  |  |  |
| No | 37 (30.3%) | 35 (94.6%) | 2 (5.4%) |  | — | — |  |
| Yes | 85 (69.7%) | 68 (80.0%) | 17 (20.0%) |  | 3.81 (0.88, 16.5) | 14.6 (0.98, 217) |  |
| **1^st^ biologic drug** |  |  |  | <0.001 |  |  |  |
| Dupilumab | 77 (63.1%) | 73 (94.8%) | 4 (5.2%) |  | — | — |  |
| Omalizumab | 32 (26.2%) | 22 (68.8%) | 10 (31.3%) |  | **6.59 (2.07, 21.0)** | **6.49 (1.79, 23.5)** |  |
| Mepolizumab | 12 (9.8%) | 7 (58.3%) | 5 (41.7%) |  | **10.4 (2.78, 38.6)** | **7.47 (1.52, 36.6)** |  |
| **Cluster by Allergen Test** |  |  |  | 0.193 |  |  |  |
| 1 | 79 (64.8%) | 70 (88.6%) | 9 (11.4%) |  | — | — |  |
| 2 | 27 (22.1%) | 20 (74.1%) | 7 (25.9%) |  | 2.41 (0.90, 6.46) | 2.39 (0.84, 6.77) |  |
| 3 | 16 (13.1%) | 13 (81.3%) | 3 (18.8%) |  | 1.75 (0.47, 6.48) | 2.21 (0.56, 8.65) |  |
| **ICS 1-year before biologic initiation** |  |  |  | 0.721 |  |  |  |
| No | 43 (35.2%) | 37 (86.0%) | 6 (14.0%) |  | — | — |  |
| Yes | 79 (64.8%) | 66 (83.5%) | 13 (16.5%) |  | 1.19 (0.45, 3.14) | 1.26 (0.47, 3.42) |  |
| **ICS+LABA 1-year before biologic initiation** |  |  |  | 0.368 |  |  |  |
| No | 14 (11.5%) | 13 (92.9%) | 1 (7.1%) |  | — | — |  |
| Yes | 108 (88.5%) | 90 (83.3%) | 18 (16.7%) |  | 2.45 (0.33, 18.3) | 1.88 (0.24, 14.7) |  |
| **OCS 1-year before biologic initiation** |  |  |  | 0.110 |  |  |  |
| No | 14 (11.5%) | 10 (71.4%) | 4 (28.6%) |  | — | — |  |
| Yes | 108 (88.5%) | 93 (86.1%) | 15 (13.9%) |  | 0.42 (0.14, 1.26) | **0.09 (0.02, 0.41)** |  |
|  |  |  |  |  |  |  |  |
| **SAE 1-year before biologic initiation** |  |  |  | 0.007 |  |  |  |
| No | 67 (54.9%) | 62 (92.5%) | 5 (7.5%) |  | — | — |  |
| Yes | 55 (45.1%) | 41 (74.5%) | 14 (25.5%) |  | **3.73 (1.34, 10.4)** | **3.15 (1.09, 9.06)** |  |
| **SAEs 1-year before biologics** |  |  |  |  |  |  |  |
| Mean (SD) | 0.94 (1.67) | 0.63 (0.92) | 2.63 (3.24) |  | **1.49 (1.29, 1.73)** | **1.39 (1.14, 1.69)** |  |
| **BMI value within 1-yr of biologics** |  |  |  |  |  |  |  |
| Mean (SD) | 24 (8) | 24 (8) | 24 (8) |  | 0.99 (0.93, 1.05) | 1.01 (0.95, 1.08) |  |
| **BMI Category within 1-yr of biologics** |  |  |  | 0.931 |  |  |  |
| Underweight | 37 (30.3%) | 31 (83.8%) | 6 (16.2%) |  | — | — |  |
| Healthy Weight | 40 (32.8%) | 34 (85.0%) | 6 (15.0%) |  | 0.91 (0.30, 2.84) | 0.96 (0.30, 3.09) |  |
| Overweight | 18 (14.8%) | 16 (88.9%) | 2 (11.1%) |  | 0.64 (0.13, 3.16) | 0.89 (0.16, 5.05) |  |
| Obesity | 27 (22.1%) | 22 (81.5%) | 5 (18.5%) |  | 1.09 (0.33, 3.56) | 1.74 (0.41, 7.42) |  |
| **OCS 1-month before biologic initiation** |  |  |  | 0.139 |  |  |  |
| No | 57 (46.7%) | 51 (89.5%) | 6 (10.5%) |  | — | — |  |
| Yes | 65 (53.3%) | 52 (80.0%) | 13 (20.0%) |  | 2.05 (0.78, 5.38) | 1.91 (0.72, 5.10) |  |
| **SABA 1-month before biologic initiation** |  |  |  | 0.296 |  |  |  |
| No | 15 (12.3%) | 14 (93.3%) | 1 (6.7%) |  | — | — |  |
| Yes | 107 (87.7%) | 89 (83.2%) | 18 (16.8%) |  | 2.80 (0,37, 20.9) | 2.43 (0.32, 18.5) |  |
| **Absolute eosinophil at baseline** |  |  |  |  |  |  |  |
| Mean (SD) | 0.50 (0.32) | 0.50 (0.28) | 0.56 (0.47) |  | 1.69 (0.47, 6.03) | 1.54 (0.45, 5.30) |  |
| **Absolute neutrophil at baseline** |  |  |  |  |  |  |  |
| Mean (SD) | 5.02 (2.32) | 4.80 (2.17) | 6.17 (2.81) |  | **1.19 (1.02, 1.39)** | **1.19 (1.02, 1.39)** |  |
| **ACT score at baseline** |  |  |  |  |  |  |  |
| Mean (SD) | 16.7 (3.8) | 16.8 (4.0) | 15.9 (2.1) |  | 0.94 (0.84, 1.05) | 0.93 (0.82, 1.05) |  |
| **Eosinophil (%) at baseline** |  |  |  |  |  |  |  |
| Mean (SD) | 6.1 (3.9) | 6.1 (3.8) | 5.9 (4.5) |  | 0.99 (0.88, 1.11) | 0.99 (0.88, 1.11) |  |
| **FEF_25-75_ predicted (%) at baseline** |  |  |  |  |  |  |  |
| Mean (SD) | 65 (26) | 67 (27) | 59 (18) |  | 0.99 (0.97, 1.01) | 0.99 (0.97, 1.101) |  |
| **FEV_1_/FVC predicted (%) at baseline** |  |  |  |  |  |  |  |
| Mean (SD) | 88.65 (3.02) | 88.92 (1.99) | 87.18 (6.02) |  | 0.91 (0.84, 0.98) | 0.92 (0.85, 1.00) |  |
| **FEV_1_ predicted (%) at baseline** |  |  |  |  |  |  |  |
| Mean (SD) | 88 (18) | 88 (19) | 88 (15) |  | 1.00 (0.97, 1.02) | 1.00 (0.98, 1.03) |  |
| **Neutrophil (%) at baseline** |  |  |  |  |  |  |  |
| Mean (SD) | 55 (13) | 54 (13) | 60 (15) |  | 1.69 (0.47, 6.03) | 1.02 (0.99, 1.05) |  |
| **(log) Total IgE** |  |  |  |  |  |  |  |
| Mean (SD) | 6.35 (1.40) | 6.36 (1.39) | 6.28 (1.48) |  | 0.95 (0.69, 1.32) | 0.93 (0.67, 1.29) |  |

Adjusted models include PDM score, biologic agent, number of SAEs in the year prior to first biologic exposure, and baseline predicted FEV_1_/FVC (%)

†The p-value is from the log-rank test for comparing survival curve across the strata of each categorical variable.

**Supplemental Table 6. Sensitivity analysis: Prediction model performance in different study sample subsets defined by history of SAE, OCS use, and biologic treatment type and duration of treatment.**

|  | **Performance** | | | | | | |
| --- | --- | --- | --- | --- | --- | --- | --- |
| **Subgroups** | **Accuracy** | **Sensitivity** | **Specificity** | **Pos Pred Value** | **Neg Pred Value** | **AUC** | **Cut-off probability** |
| **SAE 1-year history** | 0.89 | 0.79 | 0.92 | 0.79 | 0.92 | 0.91 | 0.38 |
| **Dupilumab** | 0.93 | 0.50 | 0.96 | 0.40 | 0.97 | 0.66 | 0.11 |
| **Omalizumab**† | 0.68 | 0.80 | 0.62 | 0.50 | 0.87 | 0.75 | 0.22 |
| **Mepolizumab**† | 0.75 | 0.80 | 0.71 | 0.67 | 0.83 | 0.77 | 0.31 |
| **Continued Biologic in 2^nd^ year** | 0.87 | 0.88 | 0.86 | 0.54 | 0.97 | 0.83 | 0.22 |

†Omalizumab and Mepolizumab are based on a crude model due to the small sample size of treated patients.
